# What Static Connectivity Misses: Dynamic Alpha-Band Brain Network States in First-Episode Psychosis

**DOI:** 10.1007/s10548-026-01249-9

**Published:** 2026-09-11

**Authors:** Romain Aubonnet, Mahmoud Hassan, Paolo Gargiulo, Stefano Seri, Giorgio Di Lorenzo

**Affiliations:** 1 Laboratory of Psychophysiology and Cognitive Neuroscience, Department of Systems Medicine, University of Rome Tor Vergata, Rome, Italy; 2 Institute of Biomedical and Neural Engineering, Reykjavik University, Reykjavik, Iceland; 3 MINDIG, 35000 Rennes, France; 4 Department of Science, Landspitali University Hospital, 105 Reykjavik, Iceland; 5 College of Health and Life Sciences, Institute of Health and Neurodevelopment, Aston University, Birmingham, UK; 6 Department of Clinical Neurophysiology, Birmingham Women’s and Children’s NHS FT, Birmingham, UK; 7 IRCCS Fondazione Santa Lucia, Rome, Italy

**Keywords:** First-episode psychosis, Resting-state EEG, Brain network states, Dynamic connectivity, Alpha band, Cognition

## Abstract

This study investigates resting-state EEG alpha-band connectivity in first-episode psychosis (FEP) using a novel dynamic connectivity pipeline and examines its relationship with cognitive functioning and psychopathological scores. Data from 78 individuals with FEP and 60 healthy controls (CTR) were analyzed. Source estimation was performed using eLORETA, and connectivity was quantified with the weighted phase-lag index. Static connectivity matrices were assessed using graph theory and edge-wise metrics. Dynamic connectivity matrices were clustered into five distinct brain network states (BNS) using a modified k-means algorithm, from which temporal and graph theory metrics were extracted. Static connectivity metrics revealed lower alpha connectivity in FEP than in controls. The dynamic approach identified reduced variability in the characteristic path length within the default mode network–associated BNS 1 in FEP, suggesting diminished adaptive modulation of functional integration. Subgroup analysis by medication status uncovered distinct BNS signatures for medicated and unmedicated FEP. BNS metrics were correlated with social cognition measures in CTR and with positive formal thought disorder in FEP, whereas static metrics showed no such associations. These findings suggest that relative to static connectivity metrics, dynamic connectivity provides non-redundant information and underscores the impact of medication on neural dynamics in psychosis.

**Supplementary Information:**

The online version contains supplementary material available at https://doi.org/10.1007/s10548-026-01249-9.

## Introduction

Psychosis is a multifaceted mental condition characterized by significant disruptions in the perception and cognition of reality, often manifesting through delusions, hallucinations, disorganized thought and speech, self-disturbances, and cognitive and negative symptoms, particularly in primary or idiopathic psychoses such as schizophrenia (Maj et al. 2021; Lieberman and First 2018; Jauhar et al. 2022). Functional neuroimaging research has consistently shown that psychotic disorders are associated with altered patterns of brain function, both at rest and during cognitive tasks (see (Voineskos et al. 2024) for a critical review).

Despite extensive research, inter-individual variability in patterns of brain function has posed a significant obstacle to developing a unifying model that captures the neural mechanisms underlying psychosis (Gur et al. 2007; Scheepers et al. 2018; Ganella et al. 2018; McCutcheon et al. 2020), limiting the development of targeted diagnostic tools and the early identification of effective treatments.

Among functional neuroimaging techniques, electroencephalography (EEG) offers advantageous spatiotemporal properties for correlating recurring activity patterns with their underlying functional properties, contributing to the characterization of what are now termed brain states (Biasiucci et al. 2019; Greene et al. 2023; Foster and Scheinost 2024). Its unsurpassed temporal resolution has proven particularly valuable for studying intermediate phenotypes in psychosis (Wang et al. 2022; Sponheim et al. 2000; Kam et al. 2013). Previous studies have reported abnormalities in brain electrical activity in psychotic disorders, including decreased activity in the alpha frequency band (Clementz et al. 1994; Murphy and Öngür 2019). EEG analysis techniques have more recently focused on patterns of functional brain connectivity (Hassan and Wendling 2018), and studies in schizophrenia have reported disrupted alpha-band connectivity (Di Lorenzo et al. 2015). Magnetoencephalography (MEG) has confirmed network differences between psychosis and controls in the alpha band (Phalen et al. 2020; Lottman et al. 2019) and demonstrated that specific network features are associated with distinct psychopathological domains (Phalen et al. 2020).

Psychosis is characterized by large-scale, complex functional disconnections. However, traditional static connectivity measures, often combined with graph theory metrics, may not adequately capture the complex and temporally evolving nature of brain activity (Damaraju et al. 2014; Cattarinussi et al. 2024). To better characterize the rapidly changing neural dynamics recorded with M/EEG, measures of dynamic connectivity and novel methods for modeling transient states of brain networks have been introduced (O’Neill et al. 2018; Tabbal et al. 2021; Kabbara et al. 2017; Vidaurre et al. 2017; Vidaurre et al. 2018a, b). The comparison of dynamic and static connectivity at rest in psychosis has been applied mainly to fMRI studies, which have shown that alterations observable in static connectivity can also be detected dynamically, and that subtle connectivity abnormalities that are difficult to observe at the static level become apparent using dynamic approaches (Damaraju et al. 2014; Cattarinussi et al. 2024), confirming the value of pursuing dynamic methods (Zalesky et al. 2014). Several recent EEG-based dynamic connectivity approaches have been proposed to classify psychopathology, including psychotic and non-psychotic depression or schizophrenia (Chen et al. 2024; Shen et al. 2023) or neurological disorders (Mirzaei and Ghasemi 2021). However, many of these methodologies rely on electrode-wise connectivity and therefore depend on channel density and are vulnerable to volume conduction effects—a well-established limitation of scalp-level connectivity (Schoffelen and Gross 2009). Source localization also has inherent limitations, including the non-uniqueness of the inverse problem, dependence on modeling assumptions, and residual spatial leakage between reconstructed sources. Moreover, there is a lack of consensus regarding dynamic connectivity workflows, as well as a scarcity of direct group-level comparisons or correlations with clinical scores based on dynamic features. In this context, the present study aims to contribute to this methodological gap by implementing a fully specified dynamic connectivity pipeline that combines source-level connectivity estimation, clustering-based identification of brain network states, and integration with graph-theoretical and clinical measures. By applying this framework to group-level comparisons and symptom correlations, we aim to provide a more comprehensive and reproducible approach to studying dynamic alterations in brain networks in psychosis.

This work is the first to analyze the dynamic connectivity of estimated cortical sources in resting-state EEG in psychosis. We investigated differences in source-space resting-state EEG (rsEEG) alpha-band functional connectivity patterns between individuals with first-episode psychosis (FEP) and healthy controls (CTR) using a novel methodology that integrates source localization techniques with dynamic functional connectivity and advanced clustering algorithms. Inspired by the classical concept of EEG microstates—which have been used to capture the dynamic aspects of brain electrical activity (Michel and Koenig 2018; Zanesco 2024), this approach identifies and characterizes distinct brain network states (BNS). This method has recently been successfully applied to extract EEG dynamic connectivity patterns (Duprez et al. 2022; Aubonnet et al. 2023) and has shown promising initial results in other clinical contexts (Tewarie et al. 2019; Vidaurre et al. 2016; Pescaglia et al. 2026).

To evaluate the potential advantages and the non-redundant information provided by this method relative to classical approaches, we compared results from BNS with those from static connectivity approaches based on graph theory metrics and edgewise indices. We hypothesized that dynamic connectivity would provide supplementary information beyond static connectivity, owing to its greater temporal granularity.

Furthermore, we explored the potential relationships between these dynamic network states and measures of cognitive performance and psychopathological domains. We hypothesized that BNS features would be related to cognition in both groups and, specifically, to negative symptoms in psychosis. Finally, to assess the potential effects of antipsychotic medications on brain activity (Zandstra et al. 2023; Ahnaou et al. 2017; Mackintosh et al. 2021), we investigated differences in dynamic and static alpha connectivity between medicated and unmedicated FEP individuals and their relationships with cognition and psychopathological symptoms.

## Materials and Methods

### Participants

Resting-state EEG data and the associated cognitive and behavioral metadata were obtained from the OpenNeuro database (Salisbury et al. 2022a, b) (retrieved on November 29, 2023). The dataset comprised 78 individuals with FEP (23 female; mean age 22.9 ± 4.8 years) and 60 age-matched CTR (26 female; mean age 22.8 ± 4.9 years). Cognitive performance was assessed using the MATRICS Consensus Cognitive Battery (MCCB), which evaluates Attention/Vigilance, Speed of Processing, Working Memory, Verbal Learning, Visual Learning, Reasoning and Problem Solving, Social Cognition, and an Overall composite score. The Wechsler Abbreviated Scale of Intelligence (WASI) was used to assess IQ, Vocabulary, and Matrix Reasoning.

For the FEP group, psychopathological symptoms were assessed using three instruments: (1) the Scale for the Assessment of Positive Symptoms (SAPS), comprising Hallucinations (HALL), Delusions (DELU), Bizarre Behavior (BIZB), and Positive Formal Thought Disorder (PFTD); (2) the Scale for the Assessment of Negative Symptoms (SANS), comprising Affective Flattening or Blunting (AFB), Alogia (ALO), Avolition-Apathy (AVA), Anhedonia-Asociality (ANA), and Attention (ATT); and (3) the Global Assessment Scale (GAS).

Complete demographic, clinical, and cognitive data are presented in the supplementary material (Table S1). The FEP group was further subdivided into medicated (n = 51) and unmedicated (n = 27) subgroups. Demographic and clinical characteristics of these subgroups are reported in the supplementary material (Table S2).

### EEG Recordings

For each participant, a 5-min rsEEG was recorded with eyes open. EEG data were collected using the EEG channels of an Elekta Neuromag Vectorview MEG system with a 10–10 system 60-channel cap at a sampling frequency of 1000 Hz. Because the data were available in two different EEG sensor arrays (list of sensors reported in supplementary material), we ran a validation analysis using repeated measures ANOVA based on the ROIs’ absolute power spectral density (Welch’s method) in the alpha band (7–13 Hz), with results reported in the supplementary material (Table S3). This analysis confirmed that the different arrays did not influence the ROIs estimation.

### Analysis Pipeline

**Fig. 1:**
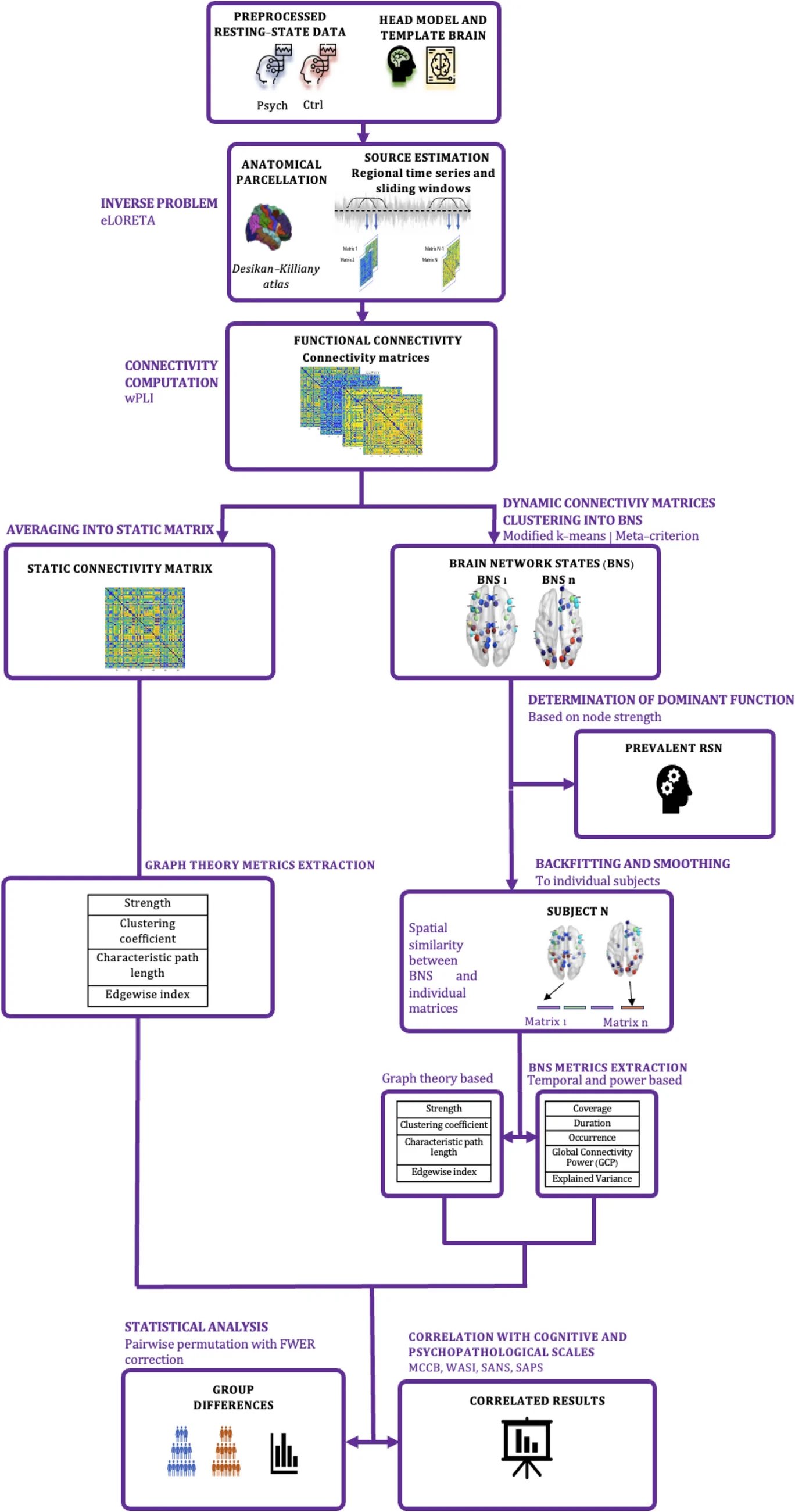
Graphical abstract of the analysis pipeline. From the preprocessed datasets, source estimation is performed, and dynamic connectivity matrices are computed in the alpha band. On the one hand, these matrices are averaged into a single static matrix per subject, from which graph theory and edge-wise metrics are computed. On the other side, the dynamic connectivity matrices are concatenated across subjects and clustered using a modified k-means algorithm into distinct brain network states (BNS). The function of each BNS is defined based on node strength. Backfitting is then performed on each subject, and smoothing is applied to remove spurious short sequences. Graph theory, temporal, and power-based metrics (derived from the microstate approach) are extracted from each subject’s final BNS sequence. All static and dynamic metrics are then statistically analyzed using permutation-based pairwise comparisons (corrected for the number of BNS) and correlation analyses with cognitive and psychopathological scales

Figure 1 provides a graphical overview of the methodology detailed in the following sections.

#### Preprocessing

Preprocessing was performed using the EEGLAB toolbox (Delorme and Makeig 2004), following standard guidelines (Li et al. 2020) and default settings. A 1-Hz high-pass filter was applied. Flat channels were removed using a flatline criterion of 5 s, and powerline noise (60 Hz and harmonics) was attenuated with a line noise criterion of 5 (De Cheveigné 2020; Klug and Kloosterman 2022). Channels with low correlation to their neighbors (< 0.6) were also removed. Artifact Subspace Reconstruction (ASR) was then applied to correct non-periodic noise (Kothe and Makeig 2013).

Subsequently, ICA decomposition was performed using the PICARD algorithm (Ablin et al. 2018; Frank et al. 2022). Independent components were labeled using the ICLabel procedure (Pion-Tonachini et al. 2019), and components classified as non-brain activity were rejected. Removed channels were interpolated using spherical interpolation based on their original locations, and the data were re-referenced to the average reference.

A second ICA decomposition, component labeling, and rejection step was then performed to remove residual periodic non-brain artifacts. The resulting preprocessed data were used for all subsequent analyses.

#### Source Estimation

Cortical time series were reconstructed from scalp EEG. According to the equivalent current dipole model, the signals recorded at M electrodes can be expressed as the linear sum of P time-varying cortical sources:

1 * X(t) = G S(t) + N(t) *

where X(t) denotes the scalp EEG signals, S(t) the estimated cortical sources, N(t) the additive noise, and G an M × P lead field matrix describing the spatial projection of each source onto the sensors, accounting for the head’s geometrical and conductive properties. The lead field matrix was derived from a three-shell head model, aligned with a standard template MRI, using the OpenMEEG plugin in Brainstorm (Gramfort et al. 2010; Tadel et al. 2011).

The EEG inverse problem aims to determine the parameters of sources at the cortical level, reducing the problem to:

2 * S(t) = W X(t) *

where W represents the inverse operator. The inverse model was calculated using exact Low Resolution Electromagnetic Tomography (eLORETA; Pascual-Marqui et al. 2011), a radially weighted minimum-norm solution designed to reduce depth bias while ensuring zero-error localization.

The reconstructed cortical activity comprised 15,000 sources, whose signals were subsequently averaged into 68 regions of interest (ROIs) defined according to the Desikan–Killiany cortical atlas (Desikan et al. 2006).

#### Connectivity Computation

The analysis was conducted in the alpha frequency band (7–13 Hz), consistent with previous studies demonstrating alpha-band alterations in EEG spectral (Clementz et al. 1994; Murphy and Öngür 2019) and connectivity (Di Lorenzo et al. 2015) features in psychosis, and with MEG results from the same patient cohort indicating distinct networks in this frequency band (Phalen et al. 2020). The alpha rhythm is of particular interest in psychosis research because of its central role in large-scale cortical network coordination, sensory gating, and top-down attentional modulation (Klimesch 2012; Jensen et al. 2024)—processes known to be disrupted in psychotic disorders.

Connectivity was quantified using the weighted phase-lag index (wPLI). This measure is robust to volume conduction and field spread (Vinck et al. 2011). This property makes it particularly suitable for EEG source-space connectivity analyses, where residual spatial leakage may inflate zero-phase-lag correlations. A sliding-window approach was adopted (window size: 600 ms, corresponding to approximately six alpha-frequency cycles as recommended by Lachaux et al. (2000), with 50% overlap) to capture the temporal evolution of connectivity patterns while ensuring reliable phase estimation (O’Neill et al. 2018; Allouch et al. 2023). This procedure resulted, for each subject, in a dynamic connectivity tensor of *nROIs* × *nROIs* × *nWindows*, where *nROIs* = 68 and *nWindows* = 979 (68 × 68 × 979). To derive static connectivity, the tensor was averaged across all time windows to produce a single *nROIs* × *nROIs* (68 × 68) matrix.

#### Static Connectivity Metrics

A preliminary Network-Based Statistic (NBS) analysis (Zalesky et al. 2010) was conducted using the static matrices, with the following parameters: t-test, threshold of 2.5, 50,000 permutations, and FDR correction. It was performed as an initial exploratory analysis to assess group-level differences in static functional connectivity before the dynamic analyses, and the results are reported in Figure S1. It was also used to compute an edgewise index (EdgeNI) following previous work by Yassine et al. (2022).

From the graph theory framework, the following metrics were computed: mean node strength, mean clustering coefficient, and characteristic path length (CPL). Definitions of each metric are provided in the supplementary material.

#### Brain Network States Clustering

Following the principles of EEG microstate analysis, clustering was performed using a modified k-means algorithm (Pascual-Marqui et al. 1995; Poulsen et al. 2018). This technique was chosen for its demonstrated ability to identify stable and recurring patterns of brain activity, as widely established in classical microstate analyses (Nagabhushan Kalburgi et al. 2023; Tarailis et al. 2024); the resulting clusters are referred to as brain network states (BNS) (Duprez et al. 2022; Aubonnet et al. 2023; Pescaglia et al. 2026).

Clustering was conducted at the group level rather than the individual level. Each subject’s dynamic connectivity matrices, being symmetric, were vectorized by extracting the lower triangular part (resulting in 2278 elements for a 68 × 68 matrix), producing vectors of dimensions 2278 × 979 per subject. These vectors were concatenated across all 138 subjects, and the unified dataset served as input for the clustering algorithm, following the methodology of Duprez et al. (2022).

Initially, solutions were explored iteratively for k = 2 to 20, with 100 restarts and 1000 iterations each. To identify the optimal number of clusters, a meta-criterion based on 11 clustering validity indices (Poulsen et al. 2018; José-García and Gómez-Flores 2023) was designed following the approach of Custo et al. (2017). The list of indices and the computation of the meta-criterion are described in the supplementary material (Table S4). Based on converging results, the search was narrowed to k = 3 to 7, with 200 restarts and 1000 iterations. The solution with the best meta-criterion score was selected as the optimal solution (see Figure S11 in the supplementary material).

#### Smoothing and Backfitting

Following BNS identification, backfitting was applied to each individual based on global map dissimilarity (GMD) (Murray et al. 2008), a topography-based and signal-invariant distance measure (Poulsen et al. 2018). Label smoothing was then performed using a small-segment rejection procedure: segments lasting fewer than five consecutive time windows were considered unstable and reassigned to the neighboring state with the highest spatial similarity. The threshold of five windows was chosen to reduce spurious, short-lived state fluctuations while preserving meaningful temporal dynamics, in line with standard practices in microstate analysis.

#### BNS Metrics and Function

##### Microstate-Derived Metrics

The following metrics were computed: global connectivity power (GCP), defined as the standard deviation of all connectivity indices at a given time point, providing a measure of connectivity strength analogous to global field power (GFP) (Murray et al. 2008); individual explained variance (iEV), the proportion of GCP explained by each BNS, indicating how well each state represents the data; total explained variance, the proportion of GCP explained by all BNS combined; coverage, the proportion of recording time during which a BNS is the dominant active state; duration, the average time a BNS remains active before transitioning to another state; and occurrence, the number of distinct episodes in which a specific BNS appears (Nagabhushan Kalburgi et al. 2023). GCP and iEV characterize the connectivity strength and representativeness of each BNS, whereas coverage, duration, and occurrence capture the temporal dynamics of network state transitions.

##### Graph Theory Metrics

Global graph theory metrics were extracted from each BNS: strength (indicating overall connectivity), clustering coefficient (a marker of functional segregation), and CPL (describing functional integration). These metrics were computed for each subject's dynamic matrix, and the mean, standard deviation, and coefficient of variation were calculated across matrices within the same cluster to capture complementary aspects of network organization and provide a parsimonious description of large-scale brain network topology.

##### BNS Function

Node strength was used to associate each BNS with resting-state networks (RSNs) (Aubonnet et al. 2023) and to characterize its predominant functional role. The following RSNs were considered: default mode network (DMN), dorsal attention network (DAN), salience network (SAN), auditory network (AUD), visual network (VIS), motor network (MOT), and Other. Each node was assigned to one of the seven RSNs based on topographic criteria (supplementary material, Figure S12). RSN strength was normalized by the number of constituent nodes, and RSNs with values exceeding the mean + 1 SD were defined as prevalent.

#### Statistical Analysis

We used a multivariate permutation approach (10,000 permutations) with family-wise error rate (FWER) correction based on the maximum statistic (Crosse et al. 2024; Maris and Oostenveld 2007). Multiple comparisons were controlled across Brain Network States (BNS) within each dynamic connectivity metric by computing the maximum statistic across states at each permutation, and separate two-sample permutation-based t-tests with maximum-statistic correction were performed for each dynamic connectivity metric.

Pairwise comparisons between groups were performed with a significance threshold of α = 0.05.

The same permutation-based framework was applied to correlation analyses. For each metric, permutation testing with maximum-statistic FWER correction was performed across BNS. However, the correction was not extended across separate metrics. Consequently, the correlation analyses should be considered exploratory and interpreted cautiously, given the increased risk of false positives associated with testing multiple metrics.

Pearson correlations were conducted to examine relationships among connectivity metrics, cognitive performance (MCCB and WASI), and psychopathological symptoms (SAPS, SANS, and GAS). For psychopathological scales, zero values (indicating absence of symptoms) were excluded. The distribution of symptom presence/absence did not differ significantly between medicated and unmedicated FEP subgroups, nor did psychopathological symptom scores differ between the two subgroups, regardless of the inclusion or exclusion of zero values (supplementary material, Table S2).

To address potential confounds, correlations were computed on residuals from multiple linear regression models: we included age as a covariate for all groups and chlorpromazine equivalents for medicated FEP. Given the exploratory nature of these analyses, only correlations with a coefficient exceeding |r|> 0.5 (indicating at least a moderate effect) are reported.

To quantify the dependence structure of the analyzed variables, an exploratory principal component analysis (PCA) was performed across the 91 connectivity-derived metrics included in the statistical analyses. The first 5 principal components explained 62%, and the first 10 explained 79% of the total variance; the effective dimensionality of the metric space, estimated as the participation ratio of the eigenvalue spectrum, was 9.6. Additional details are provided in Supplementary Material (Appendix 1 and Figure S2).

Statistical analyses were performed for four comparisons: FEP vs. CTR, medicated FEP vs. CTR, unmedicated FEP vs. CTR, and medicated FEP vs. unmedicated FEP.

## Results

### FEP vs. CTR

#### Static Connectivity

**Fig. 2:**
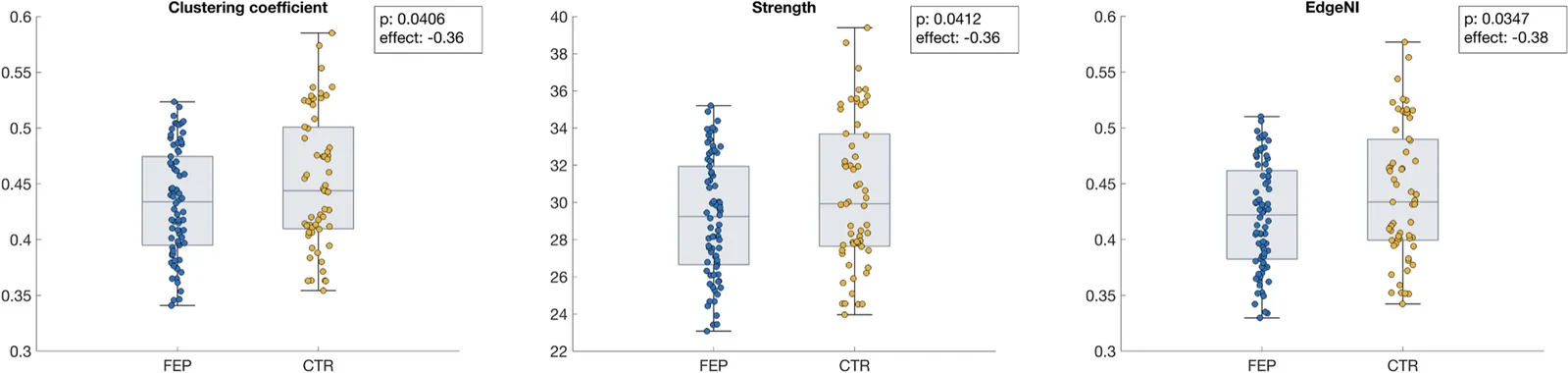
Boxplots of pairwise comparisons of significant static connectivity metrics (clustering coefficient, strength, and edgewise index) between FEP and CTR. For all three measures, FEP displays significantly lower values

FEP exhibited significantly lower clustering coefficient, strength, and edge-wise index than CTR (Fig. 2). Characteristic path length (CPL) did not differ between groups. These results indicate overall weaker local connectivity in FEP.

#### Dynamic Connectivity

##### Clustering Results

**Fig. 3:**
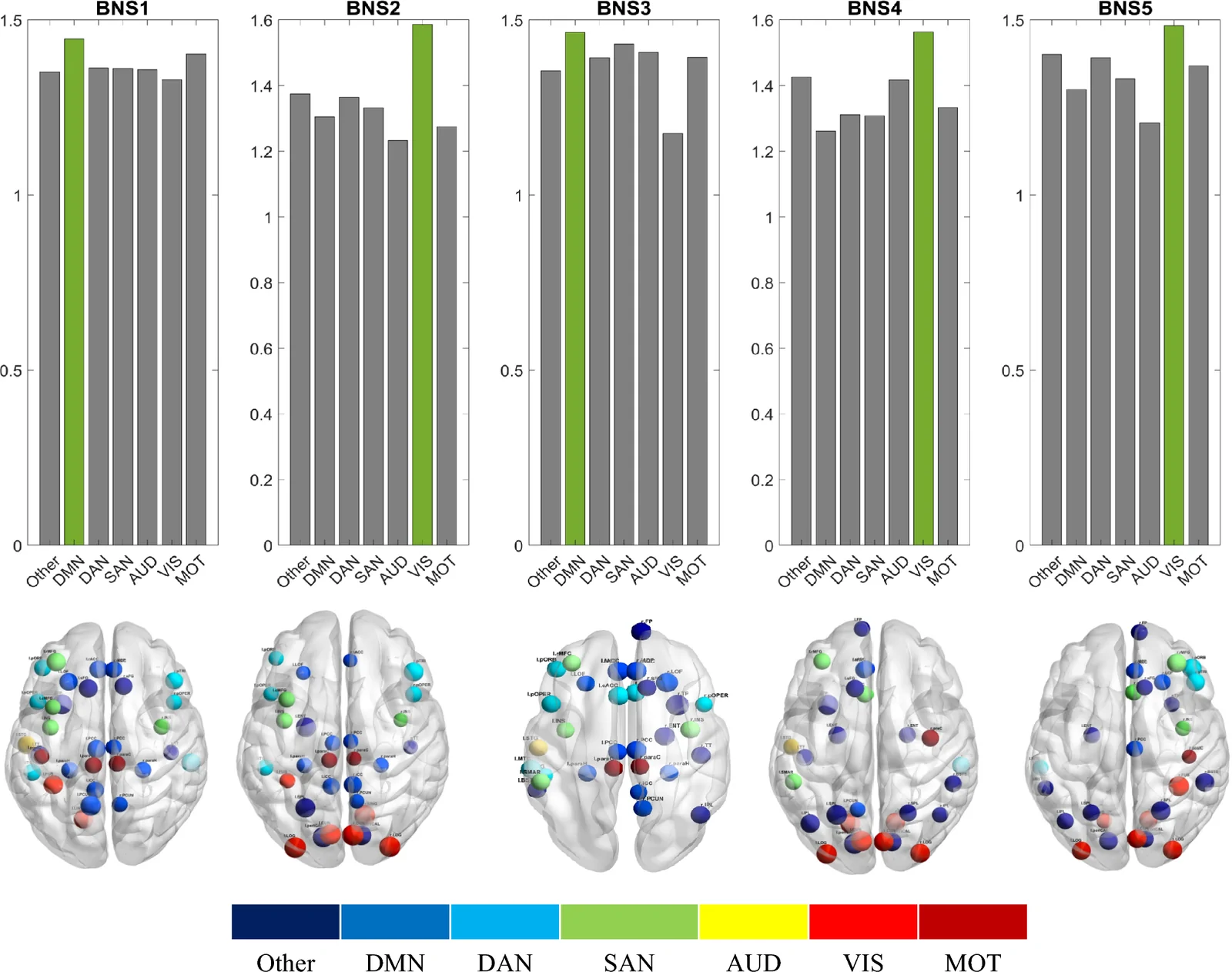
The resulting BNS, with their prevalent function and node distribution, are based on strength. BNS 1 and 3 display a stronger component in the DMN areas, while BNS 2, 4, and 5 are dominant in the visual areas

Five distinct BNS were identified from the EEG dynamic connectivity matrices. A summary of the BNS clustering results and their prevalent topography is presented in Fig. 3.

BNS 2, 4, and 5 were characterized by greater strength in visual regions. BNS 4 was lateralized to the left hemisphere, whereas BNS 5 showed right-hemisphere lateralization, particularly involving nodes from the attentional and salience networks. BNS 2 showed no significant lateralization. BNS 1 and 3 showed greater strength in DMN nodes. BNS 1 additionally exhibited left-lateralized activity in the salience and motor networks, with bilateral activation of attentional network nodes. BNS 3 showed stronger activity in “Other” RSNs in the right hemisphere but symmetric activity in the attentional and salience networks.

##### Pairwise Comparisons

**Fig. 4:**
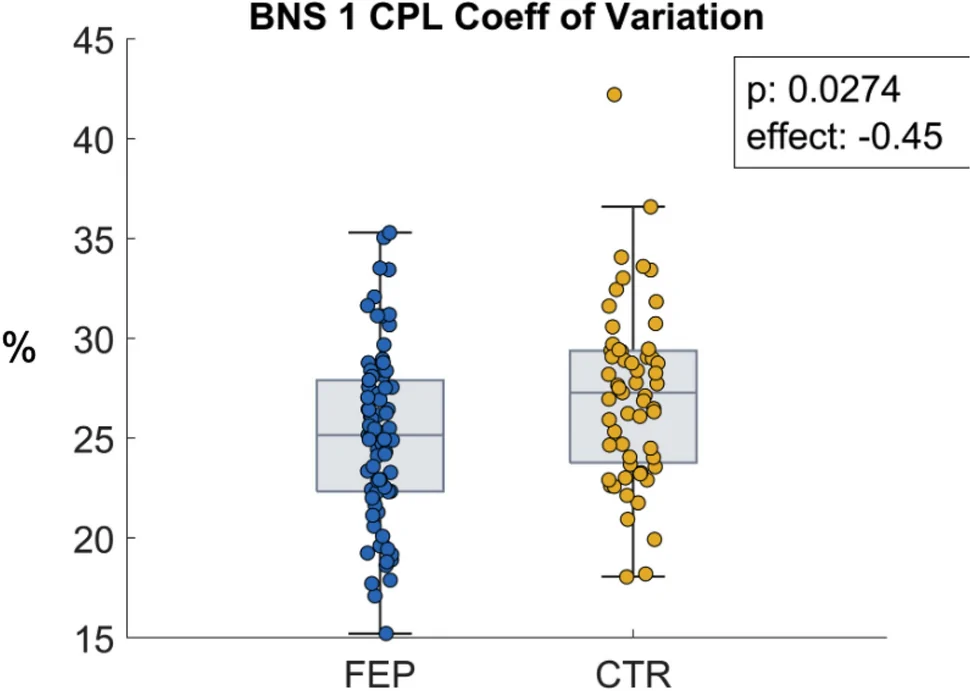
Boxplots with individual values of the significant difference in the coefficient of variation of CPL in BNS 1 between FEP and CTR

Among all BNS and graph theory metrics analyzed, the only significant difference between FEP and CTR was a lower coefficient of variation of CPL in BNS 1 for FEP (Fig. 4). This indicates that the variability of functional integration within BNS 1—a DMN-dominant state—is reduced in FEP compared with CTR, suggesting a more rigid, less adaptively modulated pattern of information transmission.

### Correlation Analysis

All correlation analyses reported in this study—both here and in the subgroup analyses (Sect. "Source Estimation")—were corrected across BNS within each metric but not across metrics; in addition, some subgroups are small (unmedicated FEP, n = 27). These analyses are therefore exploratory: they carry an increased risk of false positives and are reported as hypothesis-generating. This qualification applies to all correlations reported below and is not repeated for each individual result.

#### Static Connectivity

No significant correlations were found between static connectivity metrics and cognitive or psychopathological measures in either FEP or CTR.

#### Dynamic Connectivity

**Fig. 5:**
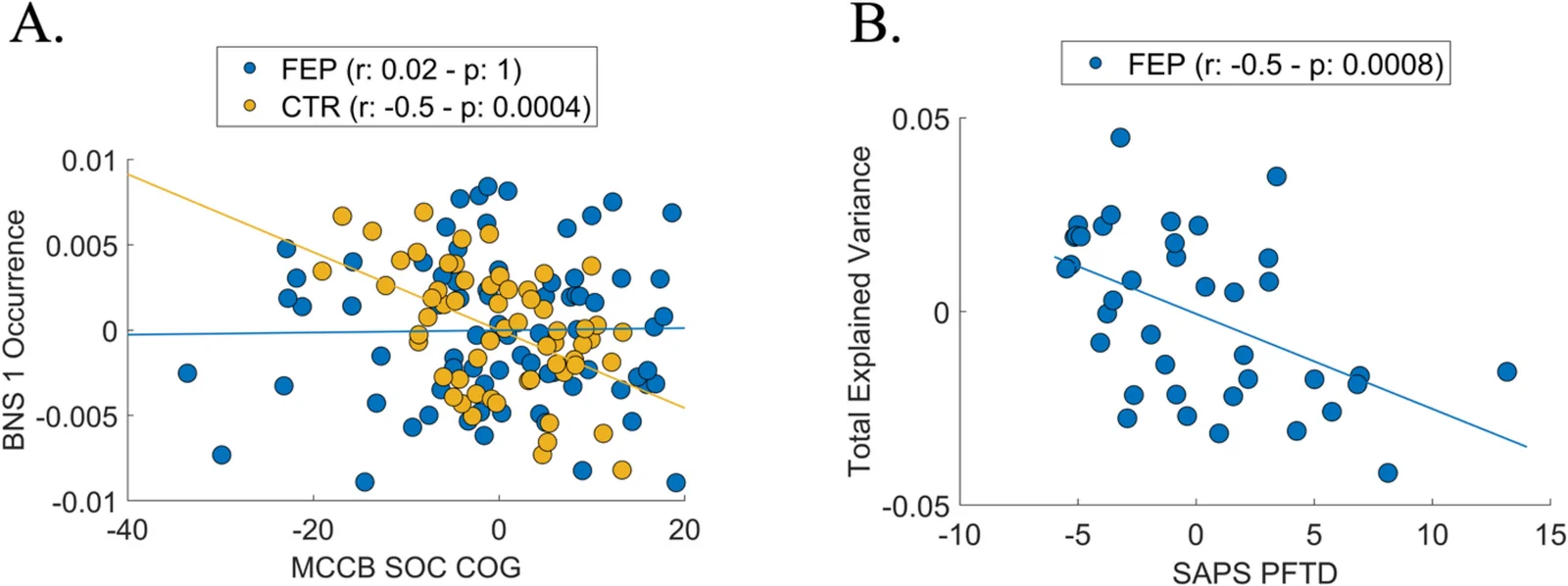
Scatter plots and correlations between BNS metrics and cognitive/psychopathological scales. (**A**) Scatter plots of the occurrence of BNS 1 and MCCB SOC COG score in CTR (yellow) and FEP (blue), showing a significant negative correlation in CTR. (**B**) Scatter plots of total explained variance and SAPS PFTD in FEP, showing a significant negative correlation

A significant negative correlation was observed between the social cognition score and the mean occurrence of BNS 1 in CTR, but not in FEP (Fig. 5a).

Regarding psychopathological domains, a significant negative correlation was observed between total explained variance and Positive Formal Thought Disorder (PFTD) scores in FEP (Fig. 5b), indicating that individuals with higher PFTD scores presented lower agreement with the group-derived BNS model.

No other connectivity-derived features were significantly correlated with cognitive or psychopathological scales.

### Subgroup Analysis: The Effect of Medication

#### Pairwise Comparisons

##### Static Connectivity

No significant differences were observed for any subgroup comparison (medicated FEP vs. CTR, unmedicated FEP vs. CTR, medicated FEP vs. unmedicated FEP).

##### Dynamic Connectivity

**Fig. 6:**
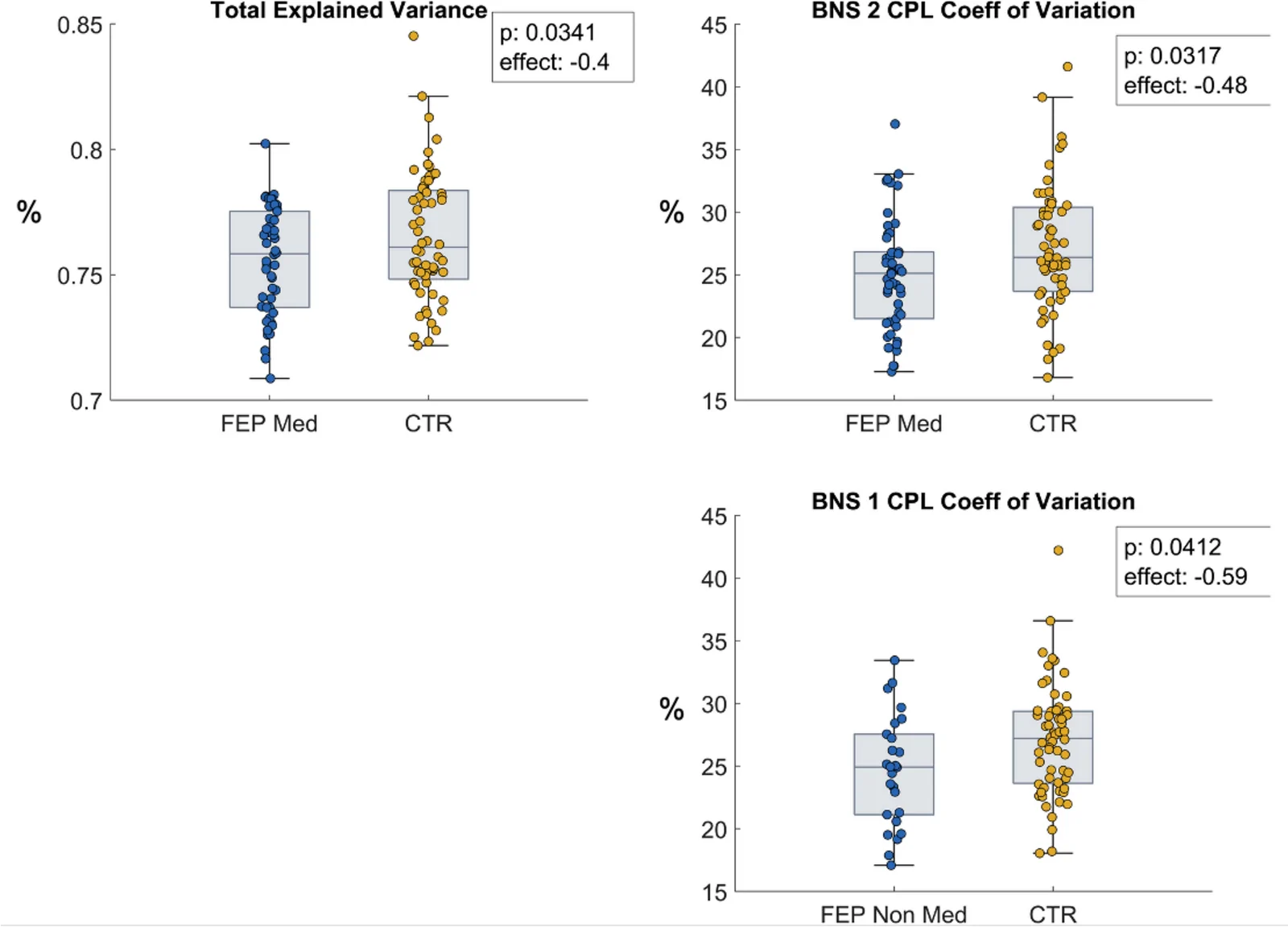
Boxplots showing subgroup pairwise comparison results. Top row: significant differences between medicated FEP and CTR (lower total explained variance and coefficient of variation of CPL in BNS 2). Bottom row: significantly lower coefficient of variation of CPL in BNS 1 for unmedicated FEP compared to CTR

Medicated FEP exhibited lower total explained variance and a lower coefficient of variation of CPL in BNS 2 compared to CTR (Fig. 6, top row). Lower total explained variance in medicated FEP indicates reduced agreement between individual connectivity patterns and the group-derived BNS model, while lower coefficient of variation of CPL reflects reduced temporal variability of this measure within BNS 2.

Unmedicated FEP showed a lower coefficient of variation of CPL in BNS 1 compared to CTR (Fig. 6, bottom row), replicating the whole-group finding. No significant differences were observed between medicated and unmedicated FEP, although significant differences relative to CTR involved different BNS in the two subgroups (BNS 2 and BNS 1, respectively).

#### Correlation Analysis

##### Cognition

**Fig. 7:**
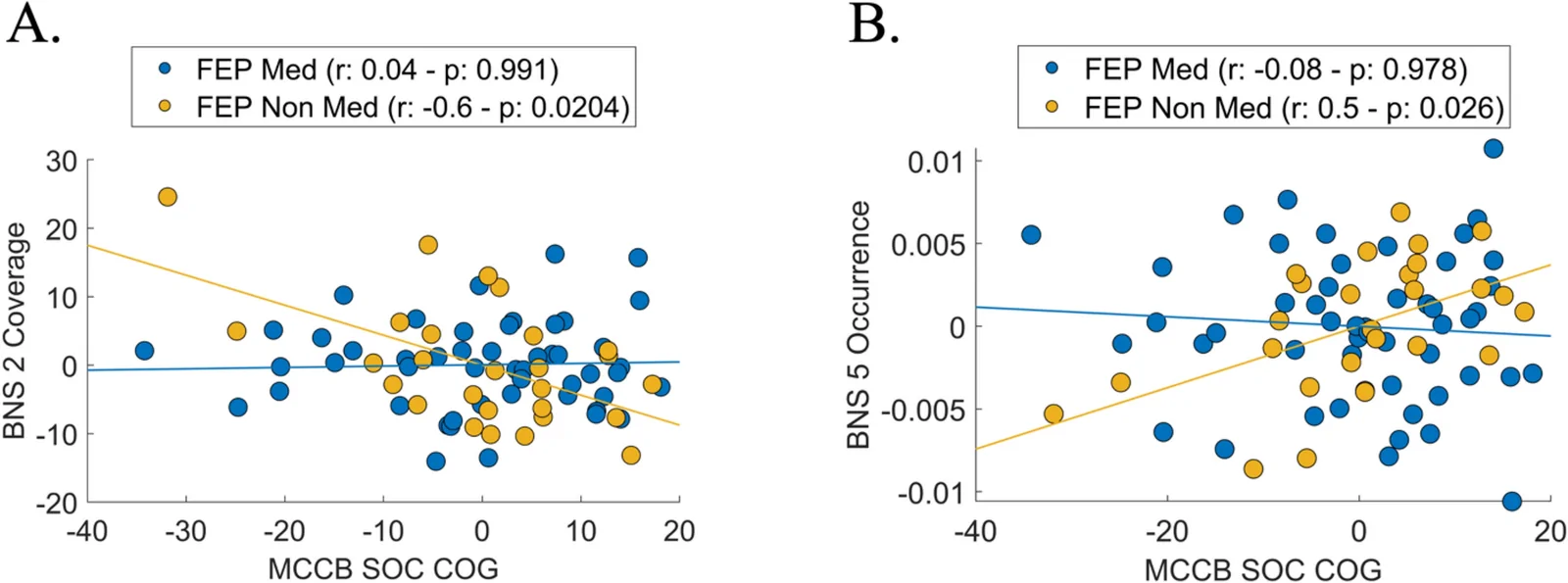
Scatter plots and correlations between BNS metrics and social cognition scores in medicated and unmedicated FEP. (**A**) Scatter plots of coverage of BNS 2 and MCCB SOC COG in unmedicated FEP (yellow) and medicated FEP (blue), showing a significant negative correlation in unmedicated FEP. (**B**) Scatter plots of the occurrence of BNS 5 and MCCB SOC COG, showing a significant positive correlation in unmedicated FEP

*Static connectivity:* No significant correlations were observed between static connectivity metrics and cognitive measures. *Dynamic connectivity:* In unmedicated FEP, coverage of BNS 2 was negatively correlated with social cognition, whereas mean occurrence of BNS 5 was positively correlated with social cognition (Fig. 7). Additional correlations between connectivity-derived measures (GCP and explained variance) and social cognition, working memory, and visual learning scores are reported in the supplementary material (Figure S3).

##### Positive Symptoms

*Static connectivity:* In medicated FEP, strength and clustering coefficient were negatively correlated, and CPL was positively correlated with PFTD. A similar pattern was observed in unmedicated FEP with bizarre behavior (BIZB) scales (supplementary material, Figure S4). *Dynamic connectivity:* Significant correlations with positive symptoms were observed, particularly with BIZB in unmedicated FEP, for connectivity-derived metrics (GCP and explained variance) and graph theory metrics across most BNS, indicating that connectivity measures across multiple states were associated with positive symptom severity in this subgroup. In BNS 5, the coefficient of variation of GCP was negatively correlated with hallucination scores in unmedicated FEP. In medicated FEP, total explained variance was negatively correlated with PFTD (supplementary material, Figures S5 and S6).

##### Negative Symptoms

**Fig. 8:**
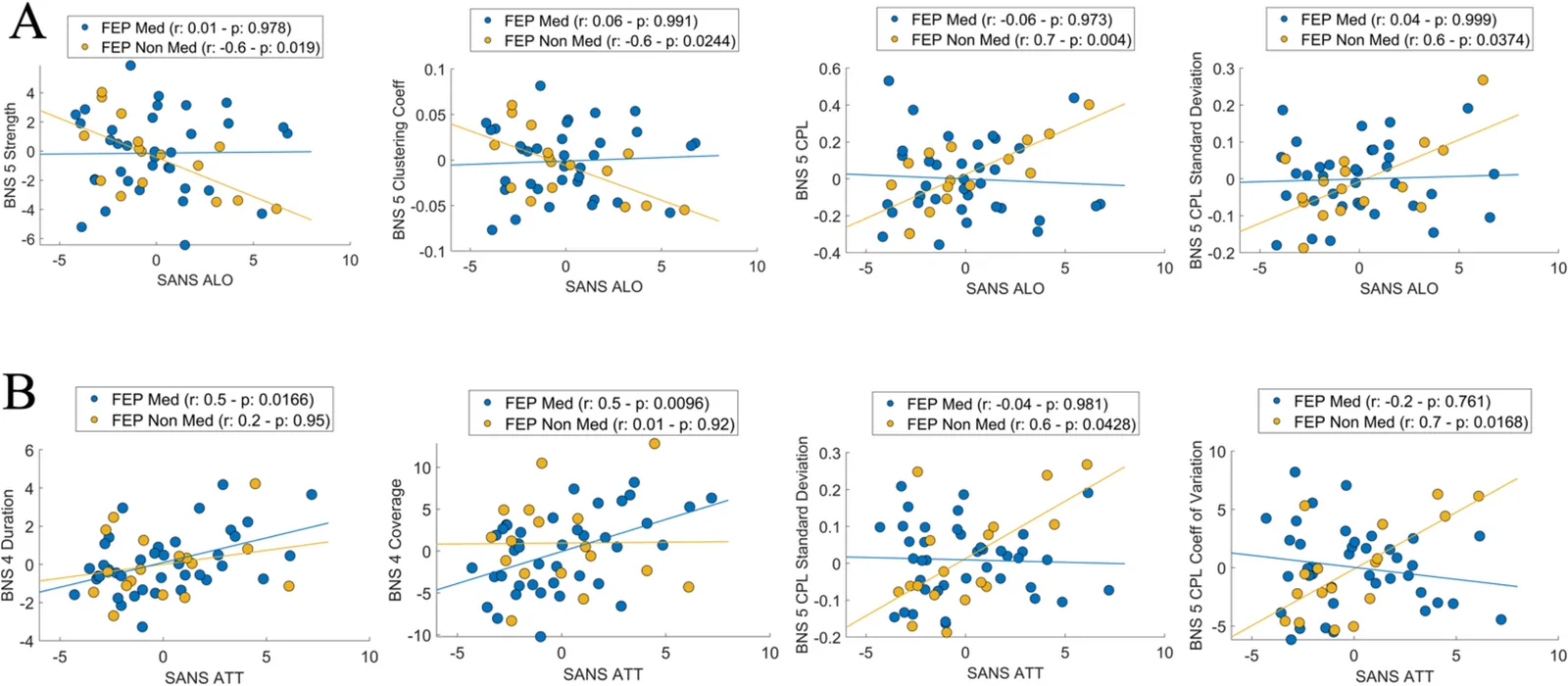
Scatter plots and correlations between BNS metrics and negative symptoms in medicated and unmedicated FEP. A: graph theory metrics in BNS 5 vs. SANS ALO in unmedicated FEP (yellow) and medicated FEP (blue). B: temporal measures in BNS 4 vs. SANS ATT, as well as graph theory metrics in BNS 5 vs. SANS ATT, with significant correlations in medicated FEP

*Static connectivity:* In unmedicated FEP, strength and clustering coefficient were negatively correlated, and CPL was positively correlated with anhedonia-asociality (ANA) and avolition-apathy (AVA) scales (supplementary material, Figure S7). *Dynamic connectivity:* The same directional pattern was observed across most BNS for both connectivity-derived and graph theory metrics, with particularly strong correlations for ANA and AVA (supplementary material, Figures S8–S10). Examining more specific symptom domains revealed that graph theory metrics of BNS 5 showed strong correlations with alogia (ALO) and attention deficit (ATT) in unmedicated FEP. In medicated FEP, temporal measures of BNS 4 were positively correlated with ATT (Fig. 8).

## Discussion

The present study evaluated whether a novel dynamic connectivity approach applied to source-reconstructed task-free EEG data in the alpha band provides non-redundant information compared to traditional static connectivity measures in individuals with first-episode psychosis. We additionally examined the potential influence of antipsychotic medication by subdividing FEP into medicated and unmedicated subgroups. Our results suggest that dynamic connectivity analysis captures features of brain network organization in FEP that are not detected by static approaches, including reduced variability in functional integration within DMN-associated network states, state-specific correlations with cognitive performance and psychopathological symptoms, and differential BNS signatures as a function of medication status. These results support the value of dynamic connectivity as a complementary framework for characterizing large-scale brain network organization and studying brain function in psychosis. However, given the limited number of robust group-level effects and the methodological and clinical heterogeneity of the sample, these findings should be interpreted with caution.

Before the individual findings are considered, it is useful to state the evidential status of each class of result. The group comparisons were assessed within a permutation framework with family-wise error correction across BNS within each metric, and constitute the primary results of this study. The correlation analyses—both at the whole-group level (Sects. "Relationship with Cognitive Performance" and "Relationship with Psychopathological Symptoms") and within the medication subgroups (Sects. "Correlation with Cognition" and "Correlation with Psychopathological Symptoms")—were evaluated with the same permutation procedure and corrected for multiple comparisons across BNS, but were not corrected across the 91 connectivity-derived metrics. They therefore carry an increased risk of false positives.

The magnitude of that risk, however, is not given by the nominal number of metrics. The exploratory PCA reported in the Supplementary Material (Appendix 1 and Figure S2) shows that the 91 metrics are strongly interdependent: the first 5 components account for 62% and the first 10 for 79% of the total variance, and the participation ratio of the eigenvalue spectrum yields an effective dimensionality of 9.6. The metric space, therefore, behaves as approximately ten independent dimensions rather than 91, so a correction that treated the metrics as independent—a Bonferroni threshold of α/91, for instance—would have been conservative by close to an order of magnitude and expected to discard true effects. Ten effective dimensions are nevertheless considerably more than one: the redundancy reduces the multiple-comparison burden without abolishing it, and the PCA therefore bounds the inflation rather than licensing its dismissal. Two limits of this estimate should also be kept in view. The participation ratio captures linear redundancy alone and describes the space of connectivity metrics; it does not account for the additional multiplicity introduced by the several cognitive and psychopathological scales against which each metric was correlated, so the effective number of tests performed exceeds the effective dimensionality reported here.

Accordingly, correlations reported below are discussed as exploratory and hypothesis-generating, and no causal claim is made. Associations are described rather than interpreted as directional or mechanistic. As partial safeguards, all reported correlations survived permutation-based correction across BNS and were additionally required to exceed |r|> 0.5. These safeguards do not substitute for correction across metrics, but they afford the least protection in the smallest subgroup: with n = 27 (unmedicated FEP). Pearson estimates are volatile, individual observations can exert disproportionate influence, and a coefficient of |r|> 0.5 is more readily reached by chance in smaller groups than in larger ones. Because this qualification applies uniformly to all correlation results, it is not repeated for each individual finding.

### Rationale for the Alpha-Band Focus

The present study focused on the alpha band (7–13 Hz), informed by a substantial body of literature demonstrating alpha-band abnormalities in psychosis. Reduced alpha power has been consistently reported in schizophrenia and first-episode psychosis (Clementz et al. 1994; Murphy and Öngür 2019), and alpha-band connectivity disruptions have been identified as a distinguishing feature of psychotic disorders (Di Lorenzo et al. 2015; Phalen et al. 2020). The alpha rhythm plays a central role in large-scale cortical network coordination, functional inhibition, sensory gating, and top-down attentional modulation (Klimesch 2012; Jensen et al. 2024; Palva and Palva 2011)—processes well established to be impaired in psychosis. Furthermore, the alpha band is particularly well-suited to dynamic connectivity methods owing to its prominence in resting-state recordings and its sensitivity to both cognitive demands and pharmacological interventions (Zandstra et al. 2023; Newson and Thiagarajan 2019). While extending this approach to other frequency bands represents an important future direction, the present focus on alpha provides a principled starting point grounded in robust prior findings.

### Dynamic Connectivity Methodology

Our dynamic connectivity pipeline comprises several well-established steps from neuroimaging and computational neuroscience: source estimation (Hassan and Wendling 2018), sliding-window connectivity computation (O’Neill et al. 2018; Schoffelen and Gross 2009), and clustering algorithms adapted from the microstate literature (Vidaurre et al. 2017, 2018a, b; Michel and Koenig 2018; Pascual-Marqui et al. 1995). This framework enables the identification of recurrent brain network states and the extraction of multiple connectivity and temporal metrics at the individual level.

In addition, the modular nature of the pipeline allows individual components (e.g., connectivity metrics, window parameters, clustering methods) to be adapted or refined in future work, which is important given the current lack of consensus regarding optimal dynamic connectivity workflows.

### Interpretation of Identified BNS

The identification of five distinct BNS provides important information about neural dynamic processing at rest (Vidaurre et al. 2017; Duprez et al. 2022; Aubonnet et al. 2023; Kabbara et al. 2021). Critically, these five states were observed in both groups without significant differences in coverage—the proportion of recording time during which a given connectivity configuration is dominant—or occurrence—the frequency with which a state is entered. This indicates that the fundamental repertoire of alpha-band connectivity configurations is preserved in FEP, and that pathology may instead manifest in the internal structure or variability of these states rather than in their gross temporal characteristics.

BNS 2, 4, and 5, predominantly associated with visual regions, confirm the significant role of visual processing in resting-state brain network activity (Clayton et al. 2018), as expected given the eyes-open recording paradigm. The hemispheric differences observed—left-lateralized activity in BNS 4 and right-lateralized activity in BNS 5—reflect functional brain lateralization (Güntürkün et al. 2020) and may represent asymmetric engagement of attentional and salience networks during rest. BNS 1 and 3, marked by predominant DMN activity, confirm the well-documented involvement of internally focused thought processes during the resting state (Andrews-Hanna 2012; Buckner et al. 2008).

### Whole-Group Analysis

#### Group Differences

Static connectivity analysis revealed decreased alpha-band connectivity in FEP compared with CTR, consistent with previously reported EEG alpha-band network dysfunction in psychosis (Clementz et al. 1994; Murphy and Öngür 2019). However, the effect sizes were small, and these differences would not survive correction for multiple comparisons. The NBS analysis provided convergent, though exploratory, support for a trend toward lower alpha connectivity in FEP, highlighting the difficulty of detecting robust group-level effects in this population (Ganella et al. 2018; McCutcheon et al. 2020).

The sole significant difference in dynamic connectivity between FEP and CTR was a lower coefficient of variation of CPL in BNS 1. This finding indicates that the CPL—a measure of functional integration reflecting the efficiency of information transmission across the network—was more stable (i.e., less variable) in FEP than in CTR. Given that FEP also tended to have a higher (though non-significant) mean CPL, this pattern may suggest a less flexible or more constrained organization of functional integration within DMN-related network configurations.

In CTR, greater CPL variability may reflect adaptive modulation of functional integration—the brain dynamically adjusting its communication pathways—a flexibility that appears diminished in FEP. This interpretation is consistent with the dysconnectivity hypothesis of psychosis (Friston 1998; Stephan et al. 2009) and aligns with growing evidence that reduced network flexibility is a hallmark of psychiatric disorders (Braun et al. 2015; Mennigen et al. 2023). It extends the findings from other imaging modalities, revealing altered alpha neural dynamics in schizophrenia (Alamian et al. 2020) and globally altered network connectivity (Houck et al. 2017). Importantly, the limited number of significant group-level findings suggests that alterations in early psychosis may be subtle or difficult to capture using global comparisons. This may be further compounded by substantial clinical heterogeneity within the FEP group, including variability in symptom profiles, underlying mechanisms, and treatment status, all of which can reduce statistical power and obscure subgroup-specific effects. In this context, the absence of widespread differences should not be interpreted as evidence of preserved network function, but rather as reflecting the disorder's complexity and the limitations of group-level analyses.

Consistent with this view, dynamic differences emerged more clearly in subgroup analyses, suggesting that stratified or individualized approaches may be necessary to better characterize network alterations in psychosis.

#### Relationship with Cognitive Performance

Static analysis revealed no significant correlations with cognitive scales, whereas dynamic connectivity identified a negative correlation between BNS 1 occurrence and social cognition in CTR. This association is consistent with the well-documented relationship between DMN activity and externally directed cognition (Raichle 2015; Fox et al. 2005). The DMN has been implicated in social cognition through its role in mentalization, self-referential processing, and theory of mind (Buckner et al. 2008; Schilbach et al. 2008), yet excessive or poorly regulated DMN engagement during rest may reflect a pattern that impairs readiness for social cognitive demands.

Contrary to our hypothesis, no significant BNS–cognition correlations were observed in the FEP group as a whole. This absence may reflect disrupted connectivity patterns that decouple neural dynamics from cognitive output (McCutcheon et al. 2020) or, more likely, the heterogeneity of the FEP group, which may obscure subgroup-specific relationships. The observation that associations emerged for BNS metrics but not for static measures suggests that dynamic and static approaches may capture different aspects of brain–behavior relationships. Replication is required before conclusions regarding the relative sensitivity of the two approaches can be drawn. A more granular subgroup stratification, potentially based on biotype approaches (Clementz et al. 2016, 2020, 2022; Tang et al. 2024), could reveal meaningful BNS–cognition associations in psychosis.

#### Relationship with Psychopathological Symptoms

Contrary to our initial hypotheses, no significant associations were observed between BNS metrics and negative symptoms at the whole-group level. This lack of expected effects highlights important limitations of the present study, including limited statistical power and substantial inter-individual variability within the FEP group. It also suggests that the relationship between dynamic connectivity and clinical or cognitive features may be more complex and potentially mediated by subgroup-specific mechanisms that are not captured in a group-level analysis. Future studies using larger, more homogeneous samples or stratified approaches will be necessary to clarify these relationships.

The association with PFTD is noteworthy because formal thought disorder has been linked to heterogeneous neural correlates involving distinct brain areas depending on the specific nature of the experience (Maderthaner et al. 2023), and structural brain changes have been identified in association with formal thought disorder dimensions (Stein et al. 2022). The observation that this association involved a global measure of BNS model fit rather than a specific state may be consistent with alterations spanning multiple network configurations.

### Subgroup Analysis: Effects of Medication

Dividing FEP by medication status addressed one potential source of heterogeneity and revealed informative dissociations between medicated and unmedicated subgroups.

#### Group Differences

Notably, static connectivity analysis revealed no significant differences across subgroup comparisons, highlighting the limited sensitivity of static approaches when applied to smaller, more homogeneous samples.

In contrast, dynamic connectivity identified distinct patterns in the two groups. Medicated FEP showed lower total explained variance and a lower coefficient of variation of CPL in BNS 2 (a visual-network–dominant state) compared to CTR. The lower total explained variance may reflect greater variability in neural dynamics, while the more constrained CPL in BNS 2 suggests reduced network flexibility. This finding was not observed when comparing unmedicated FEP with CTR or the whole FEP group, hinting that antipsychotic medication may influence network dynamics in ways that produce distinct, detectable connectivity signatures. This interpretation is consistent with studies in schizophrenia and fMRI demonstrating that antipsychotic treatment modulates network topology and connectivity patterns (Liu et al. 2022; Towlson et al. 2019; Hadley et al. 2016), and with EEG/MEG evidence of pharmacological effects on resting-state connectivity (Zandstra et al. 2023; Mackintosh et al. 2021).

Unmedicated FEP showed a lower coefficient of variation of CPL in BNS 1 compared to CTR—the same finding observed in the whole-group analysis. The persistence of this BNS 1 finding in the unmedicated subgroup may indicate that altered temporal properties of functional integration are present independently of treatment status. This interpretation remains speculative, given the cross-sectional design and subgroup sample sizes. The observation that medicated and unmedicated FEP differ from CTR in distinct BNS—despite no direct differences between the two subgroups—provides a nuanced picture in which antipsychotic medication may partially normalize certain intrinsic connectivity abnormalities while introducing new, treatment-related signatures. Longitudinal studies will be necessary to validate whether these patterns reflect treatment effects, illness-related factors, or both.

#### Correlation with Cognition

Static connectivity revealed no significant correlations with cognition in any subgroup. In contrast, dynamic connectivity metrics revealed that in unmedicated FEP, temporal measures of BNS were associated with social cognition: coverage of BNS 2 was negatively correlated, and occurrence of BNS 5 was positively correlated with social cognition. Prolonged engagement in a visual-dominant connectivity state (BNS 2) may reflect a pattern of network allocation that impairs social cognitive processing, whereas frequent transitions into BNS 5—a state with right-hemisphere attentional and salience network involvement—may support social cognitive function. Social cognition has been identified as one of the cognitive domains most consistently impaired in first-episode psychosis (Green et al. 2015; Savla et al. 2013), and its neural substrates involve dynamic interactions among attentional, salience, and default mode networks (Schilbach et al. 2008; Uddin 2015). The fact that these associations emerged exclusively through dynamic metrics in the unmedicated subgroup underscores both the sensitivity of BNS-based approaches and the importance of accounting for medication effects. Replication in larger independent samples will be necessary before conclusions regarding sensitivity or medication effects can be established.

Additional correlations between connectivity-derived measures and working memory and visual learning scores were observed in the supplementary analyses, further supporting the utility of dynamic connectivity metrics for characterizing brain–behavior relationships in psychosis (Perrottelli et al. 2022).

#### Correlation with Psychopathological Symptoms

**Positive symptoms.** Both static and dynamic connectivity metrics were associated with positive symptoms, though with important differences in specificity and scope. In static connectivity, strength and clustering coefficient were negatively correlated (and CPL positively correlated) with PFTD in medicated FEP and with bizarre behavior (BIZB) in unmedicated FEP, indicating that weaker local connectivity is associated with greater positive symptom severity. Dynamic connectivity extended these findings considerably: BIZB in unmedicated FEP was correlated with connectivity-derived metrics (GCP and explained variance) and graph theory metrics across nearly all BNS, suggesting that these associations were distributed across multiple states rather than confined to a specific network configuration (Whitfield-Gabrieli et al. 2009). In BNS 5, the coefficient of variation of GCP was negatively correlated with hallucination scores in unmedicated FEP, suggesting an association between this network state and hallucination scores. The spatial distribution of the state overlaps with regions involved in visual and attentional processing, although the present results do not allow inference regarding specific neural mechanisms (Jardri et al. 2014; Allen et al. 2012). In medicated FEP, total explained variance was negatively correlated with PFTD, extending the whole-group finding and supporting the observation that reduced model fit was associated with PFTD across analyses.

**Negative symptoms.** Static connectivity metrics in unmedicated FEP were correlated with ANA and AVA, confirming the link between reduced local connectivity and more severe negative symptoms. Dynamic connectivity reproduced these associations across most BNS, with particularly strong correlations for ANA and AVA in both connectivity-derived and graph theory metrics, consistent with the view that negative symptoms are associated with widespread rather than focal connectivity deficits (Galderisi et al. 2018; Sanfratello et al. 2019).

In addition, dynamic connectivity measures showed several symptom-specific associations that were not observed using static metrics. In BNS 5, graph theory metrics showed strong correlations with alogia (ALO) and attention deficit (ATT) in unmedicated FEP. The spatial distribution of this state is consistent with evidence linking right-hemisphere function to poverty of speech and attentional performance in schizophrenia (Cavelti et al. 2018; Kircher et al. 2018). In medicated FEP, temporal measures of BNS 4 were correlated with ATT, involving a left-lateralized visual state, suggesting that medication status influences the relationship between network dynamics and attentional symptoms, although the mechanisms underlying this association remain unclear. These state- and subgroup-specific associations suggest that dynamic connectivity measures may capture symptom-specific associations, warranting further investigation in larger, more homogeneous samples.

### Methodological Considerations, Advantages, and Limitations

#### Static vs. Dynamic Connectivity: Complementary Information

Static and dynamic connectivity analyses provided partially overlapping but distinct information. At the whole-group level, static connectivity appeared more sensitive to group differences, although these effects were limited and did not survive multiple-comparison correction. In contrast, dynamic measures did not reveal widespread group differences but identified alterations in specific states and in subgroup analyses.

In correlation analyses, several relationships observed in static connectivity were also reflected in dynamic measures across multiple BNS, indicating a degree of convergence between approaches. However, dynamic connectivity revealed additional state-specific associations and relationships with cognitive measures that were not detected using static metrics. Temporal features of BNS (e.g., coverage, duration, and occurrence) provided an additional dimension not accessible in static analyses.

Overall, these findings suggest that, compared to static measures, dynamic connectivity offers complementary rather than diverging insights into brain network organization. Combining both approaches may therefore provide a more comprehensive characterization of brain network alterations, particularly in heterogeneous clinical populations.

fMRI studies have revealed that individuals with psychosis or ultra-high risk of psychosis present brain dysconnectivity affecting cognitive capacity and potentially disturbing cortical-subcortical circuitry (Jensen et al. 2024; Allen et al. 2012). While fMRI allows direct investigation of subcortical alterations, EEG-based BNS analysis could serve as a sensitive, non-invasive method for detecting cortical-level signatures linked with subcortical dysfunction.

#### Frequency Bands

As discussed above, the present work focused on the alpha band, supported by strong prior evidence. However, focusing the analysis on a single frequency band will inevitably limit the scope of the study. Extension to other frequency bands—particularly theta and beta bands, which have also been implicated in psychosis (Schmiedt et al. 2005; Uhlhaas and Singer 2010)—could provide complementary information and is an important direction for future research.

#### Methodological Variability

Several aspects of the present study design substantially constrain the interpretation and generalizability of the findings and should be explicitly considered when interpreting the results.

First, methodological variability may arise from the choice of inverse solution, connectivity metric (Allouch et al. 2023), or clustering algorithm (Jajcay and Hlinka 2023; Alonso and Vidaurre 2023), each of which may influence the specific BNS obtained.

In particular, while wPLI was chosen for its robustness to volume conduction, alternative connectivity measures (e.g., coherence-based or amplitude-based metrics) may capture different aspects of inter-regional interactions. Similarly, the choice of window length reflects a trade-off between temporal resolution and reliability of phase estimation, and different parameterizations may slightly alter the temporal structure of the identified brain network states.

Nevertheless, the methodological choices adopted here are consistent with established recommendations in the field, and the interpretation of results focuses on patterns that are consistent across multiple metrics. Future studies should assess the reproducibility of these findings across alternative connectivity measures and parameter settings.

Second, while the group-level clustering approach is advantageous for identifying common patterns, it may overlook individual variability. As a result, subject-specific network configurations may be partially averaged out, potentially reducing sensitivity to individual differences that are central to psychosis. Incorporating an individual-level clustering step could provide valuable insights, enabling the interpretation of findings from a subject-specific perspective, as has been performed in some studies (Doss et al. 2024). Third, the dataset was retrospective and sourced from a public repository, limiting control over acquisition parameters and potentially introducing variability. The lack of extended clinical and phenotypic information also limited the analysis, precluding new subgroup analyses. Relevant factors such as smoking, alcohol and caffeine consumption, and sex differences were not available in the present dataset. As these variables are known to influence EEG measures, their absence may contribute to variability in the results and should be considered when interpreting the findings.

Fourth, the cross-sectional design precludes causal inference about the relationship between BNS features and symptoms. Finally, as set out at the beginning of the Discussion, the correlation analyses were not corrected for multiple comparisons across metrics. The exploratory PCA reported there bounds the resulting inflation but does not remove it, and it should not be read as a substitute for formal correction; replication in independent samples remains the decisive test of these associations.

In future work, several aspects of the methodology could be refined to increase the granularity and robustness of the approach: more stringent subgroup definitions, comparisons between individual-level and group-level clustering, and integration with normative modeling, which is highly promising for developing patient-specific approaches (Ebadi et al. 2024). In particular, validation in independent cohorts and the use of longitudinal designs will be essential to determine the robustness and clinical relevance of the observed patterns.

The aim of the present work is not to validate dynamic connectivity measures as diagnostic biomarkers, but rather to explore their value as a complementary tool for characterizing brain network function in psychosis. The results should therefore be interpreted within a descriptive, hypothesis-generating framework: while the associations with clinical measures suggest potential relevance, they are insufficient to support diagnostic or predictive claims and require validation in independent, longitudinal datasets.

### Future Directions and Clinical Implications

In conclusion, the present study demonstrates the feasibility of applying dynamic connectivity analyses to source-space EEG data in psychosis, highlighting the potential of this approach to complement static connectivity measures.

Recent work has demonstrated that brain network states extracted from EEG correlate with corticospinal excitability during transcranial magnetic stimulation (Makkinayeri et al. 2025), further supporting the translational potential of this approach.

Moreover, the present work employed graph theory metrics and classical temporal and power-based measures derived from microstates. Recent studies have highlighted the value of studying transition probabilities within EEG microstates (Artoni et al. 2023; Jia et al. 2021), underscoring the dynamicity and complexity of brain activity at rest. Transition probability analysis applied to BNS sequences represents a promising avenue to explore, along with complexity measures that have already shown results in the current patient pool (Von Wegner et al. 2024).

Future work should aim to replicate these results in independent cohorts, extend analyses across frequency bands, and incorporate longitudinal designs to better characterize the evolution of network dynamics in psychosis. Further methodological developments, including approaches that better capture individual variability and temporal transitions between states, will be critical for advancing the utility of dynamic EEG connectivity in clinical research. The field of brain state research may prove vital for understanding the relationships among neural dynamics, physiological changes, and clinical phenotypes (Greene et al. 2023). Future work should aim to replicate these findings in independent cohorts, extend analyses across frequency bands, and incorporate longitudinal designs to better characterize the evolution of network dynamics in psychosis. Overall, dynamic connectivity should be viewed as a complementary framework for investigating brain network function in psychosis rather than as a standalone diagnostic or predictive tool.

## Supplementary Information

Below is the link to the electronic supplementary material.

Supplementary file 1

## Data Availability

The EEG data analyzed in this study are publicly available from the OpenNeuro database (https://doi.org/10.18112/OPENNEURO.DS003944.V1.0.1 and https://doi.org/10.18112/OPENNEURO.DS003947.V1.0.1). Analysis scripts are available from the corresponding author upon reasonable request.
