# Supplementary material for: What Static Connectivity Misses: Dynamic Alpha-Band Brain Network States in First-Episode Psychosis
Source: Brain Topogr. 2026 Sep 11;39(6):96. doi: 10.1007/s10548-026-01249-9 (PMC13569610; doi:10.1007/s10548-026-01249-9)
Supplement: Supplementary file 1 [file 10548_2026_1249_MOESM1_ESM.docx]

**Supplementary material**

**Figure S1.** Static connectivity differences in FEP vs. CTR.

**Appendix 1.** Exploration of the interdependency of features using PCA.

**Figure S2.** Dependence structure of the dynamic connectivity metrics

**Figure S3.** Scatter plots and correlations between BNS metrics and cognition scales in medicated and unmedicated FEP.

**Figure S4**. Scatter plots and correlations between graph theory metrics of static connectivity and positive symptoms in medicated and unmedicated FEP.

**Figure S5**. Scatter plots and correlations between BNS metrics and positive symptoms in medicated and unmedicated FEP.

**Figure S6.** Scatter plots and correlations between BNS metrics and positive symptoms scales in medicated and unmedicated FEP.

**Figure S7**. Scatter plots and correlations between graph theory metrics of static connectivity and negative symptoms in medicated and unmedicated FEP.

**Figure S8.** Scatter plots and correlations between BNS connectivity-derived metrics and negative symptoms (SANS ANA) scales in medicated and unmedicated FEP.

**Figure S9.** Scatter plots and correlations between BNS connectivity-derived metrics and negative symptoms scales (SANS AVA) in medicated and unmedicated FEP.

**Figure S10.** Scatter plots and correlations between BNS graph theory metrics and negative symptoms scales (SANS AVA) in medicated and unmedicated FEP.

**Table S1.** Descriptive and univariate analysis of sociodemographic, cognitive, and clinical variables in FEP and CTR.

**Table S2.** Descriptive and univariate analysis of sociodemographic, cognitive, and clinical variables in FEP medicated and unmedicated.

**Table S3.** Influence of channel array on the ROI spectral activity.

**Appendix 2.** Graph Theory measures

**Appendix 3.** Microstate-derived measures

**Table S4 –** List of clustering criteria for the Meta-criterion

**Appendix 4.** Meta-criterion computation

**Figure S11.** Criteria and metacriterion results applied to the cluster solution.

**Figure S12.** List of ROIs and RSN.

**Abbreviations**

BNS – Brain Network States

CPL - Characteristic path length

CTR – Control

FEP – First episode of psychosis

FDR – False Discovery Rate

GCP – Global Connectivity Power

MCCB - MATRICS Consensus Cognitive Battery

NBS – Network-Based Statistics

PCA – Principal Component Analysis

ROI – Regions of interest

RSN – Resting-state networks

SANS - Scale for the Assessment of Negative Symptoms

SAPS - Scale for the Assessment of Positive Symptoms

MCCB SOC COG – Social cognition score of the MCCB

MCCB VIS – Visual processing score of the MCCB

MCCB WM – Working memory score of the MCCB

Symptoms from SANS

AFB - Affective Flattening or Blunting

ALO – Alogia

ANA - Anhedonia-Asociality
ATT – Attention

AVA - Avolition-Apathy

Symptoms from SAPS

BIZB - Bizarre Behavior

DELU – Delusion

HALL - Hallucination
PFTD – Positive Formal Thought Disorder

**Figure S1.** Static connectivity differences in FEP vs. CTR.

The figure shows the significant edgewise connectivity differences computed using the t-test (50,000 permutations, FDR correction) in the Network-Based Statistics (NBS) toolbox. A lower connectivity is observed in the FEP group. Figure S1A shows the significant networks in NBS, and Figure S1B shows a heatmap of significant edges with their associated t-values. Figure S1C represents the significant nodes, with each node size representing the degree (number of connections) of each node. Each node color represents a specific RSN.

This analysis was also replicated for other subgroups (FEP medicated vs CTR; FEP non medicated vs CTR; FEP medicated vs FEP non medicated), and yielded no significant results.

**Appendix 1. Exploration of the interdependency of features using PCA.**

To quantify the dependence structure among the analyzed variables, an exploratory principal component analysis (PCA) was performed across the 91 connectivity-derived metrics included in the statistical analyses. Although 91 variables were analyzed, the first 5 principal components accounted for 62% of the total variance, and the first 10 for 79%; the effective dimensionality of the metric space, estimated as the participation ratio of the eigenvalue spectrum, was 9.6, suggesting substantial interdependence among the metrics. These results are shown in Figure S2.

**Figure S2. Dependence structure of the dynamic connectivity metrics**. (A) Scree plot showing the variance explained by each principal component derived from the 91 dynamic connectivity metrics included in the statistical analyses. (B) Cumulative variance explained by successive principal components. The first 10 principal components accounted for 79% of the total variance, while the effective dimensionality estimated using the participation ratio was 9.6.

A

**Figure S3.** Scatter plots and correlations between BNS metrics and cognition scales in medicated and unmedicated FEP. The top row displays the scatter plots and correlation results of the explained variance of BNS 2 and BNS 5 with the MCCB SOC COG score in unmedicated FEP (yellow) and medicated FEP (blue), with a significant correlation in unmedicated FEP. The bottom row displays the scatter plots and the correlation results of the coefficient of variation of GCP of BNS 2 and explained variance of BNS 5 with, respectively, the MCCB WM score and the MCCB VIS score in unmedicated FEP (yellow) and medicated FEP (blue), with a significant correlation in unmedicated FEP for the first figure, and medicated FEP for the second.

**Figure S4**. Scatter plots and correlations between graph theory metrics of static connectivity and positive symptoms in medicated and unmedicated FEP. The top row displays scatter plots and correlation results for static metrics and SAPS PFTD scores in unmedicated FEP (yellow) and medicated FEP (blue), with a significant correlation in medicated FEP. The bottom row displays the correlation results of static metrics and the SAPS BIZB scores in unmedicated FEP (yellow) and medicated FEP (blue), with a significant correlation in unmedicated FEP.

**Figure S5**. Scatter plots and correlations between BNS metrics and positive symptoms in medicated and unmedicated FEP. The top row displays the correlation between GCP metrics and SAPS BIZB scores in unmedicated FEP (yellow) and medicated FEP (blue), with a significant correlation in unmedicated FEP. The bottom row displays the correlation results of total explained variance metrics and the SAPS PFTD scores in unmedicated FEP (yellow) and medicated FEP (blue), with a significant correlation in medicated FEP, as well as the correlation results of the coefficient of variation of GCP in BNS 5 and the SAPS HALL scores in unmedicated FEP (yellow) and medicated FEP (blue), with a significant correlation in unmedicated FEP.

**Note.** Regarding potential outliers in SAPS BIZB, we performed an additional analysis, removing data points above (or below) 1.5 IQR points from the third (or the first) quartile of the data. The results showed a different correlation coefficient, but it remained not significant and therefore did not affect the results.

**Figure S6.** Scatter plots and correlations between BNS metrics and positive symptoms scales in medicated and unmedicated FEP. The top row displays the correlation results of CPL of BNS 2, 3, 4, and 5 with the SAPS BIZB in unmedicated FEP (yellow) and medicated FEP (blue), with a significant correlation in unmedicated FEP. The bottom row displays the correlation results for the coefficient of variation of strength and the clustering coefficient of BNS 2, and the mean strength and clustering coefficient of BNS 4, with the SAPS BIZB score in unmedicated FEP (yellow) and medicated FEP (blue), with a significant correlation in unmedicated FEP.

**Note.** Regarding potential outliers in SAPS BIZB, we performed an additional analysis, removing data points above (or below) 1.5 IQR points from the third (or the first) quartile of the data. The results showed a different correlation coefficient, but it remained not significant and therefore did not affect the results.

**Figure S7**. Scatter plots and correlations between graph theory metrics of static connectivity and negative symptoms in medicated and unmedicated FEP. The top row displays the correlation between static metrics and SANS ANA scores in unmedicated FEP (yellow) and medicated FEP (blue), with a significant correlation in unmedicated FEP. The bottom row displays the correlation between static metrics and SANS AVA scores in unmedicated FEP (yellow) and medicated FEP (blue), with a significant correlation in unmedicated FEP.

**Figure S8.** Scatter plots and correlations between BNS connectivity-derived metrics and negative symptoms scales (SANS ANA) in medicated and unmedicated FEP. The top row displays the GCP correlation results for BNS 1-3 with the SANS ANA in unmedicated FEP (yellow) and medicated FEP (blue), with a significant negative correlation in unmedicated FEP. The bottom row displays the GCP correlation results for BNS 4 and 5, along with the total explained variance, for the SANS ANA in unmedicated FEP (yellow) and medicated FEP (blue), with a significant negative correlation in unmedicated FEP.

**Figure S9.** Scatter plots and correlations between BNS connectivity-derived metrics and negative symptoms scales (SANS AVA) in medicated and unmedicated FEP. The top row displays the GCP correlation results for BNS 1-3 with the SANS ANA in unmedicated FEP (yellow) and medicated FEP (blue), with a significant negative correlation in unmedicated FEP. The bottom row displays the GCP correlation results for BNS 4 and 5, along with the total explained variance with the SANS AVA in unmedicated FEP (yellow) and medicated FEP (blue), with a significant negative correlation in unmedicated FEP.

**Figure S10.** Scatter plots and correlations between BNS graph theory metrics and negative symptoms scales in medicated and unmedicated FEP. The top row displays the correlation results for strength, clustering coefficient, and CPL of BNS 2 with the SANS AVA in unmedicated FEP (yellow) and medicated FEP (blue), with significant correlations in unmedicated FEP. The middle row displays the correlation results for strength, clustering coefficient, and CPL of BNS 3 with the SANS AVA in unmedicated FEP (yellow) and medicated FEP (blue), with significant correlations in unmedicated FEP. The bottom row displays the correlation results for strength, clustering coefficient, and CPL of BNS 4 with the SANS AVA in unmedicated FEP (yellow) and medicated FEP (blue), with significant correlations in unmedicated FEP.

**Note.** Regarding potential outliers in the CPL of BNS 3 & 4, we performed an additional analysis, removing data points above (or below) 1.5 IQR points from the third (or the first) quartile of the data. The results showed a different correlation coefficient, but it remained not significant and therefore did not affect the results.

**Table S1. Descriptive and univariate analysis of sociodemographic, cognitive, and clinical variables in FEP and CTR.** Continuous variables are reported in the format mean (range, standard deviation) and underwent a Welch’s t-test, and Hedges’g was computed. For the categorical variables, a χ^2^-test was performed.

|  | **FEP** | **CTR** | **Test-statistic** |
| --- | --- | --- | --- |
| **n** | 78 | 60 |  |
| Age (years) | 22.8 (13-36, 4.9) | 22.9 (12.8-38.3, 4.8) | t_136_ = 0.07, p = 0.94, g = 0.01 |
| Sex (F/M) | 23/55 | 26/34 | $\chi_{1}^{2}$ = 2.84, p = 0.09 |
| Race  (Asian/Black/Mixed/Undisclosed/White) | 6/25/6/1/40 | 13/6/2/0/39 | $\chi_{4}^{2}$ **= 15.15, p = 0.004** |
| Ethnicity  (Hispanic/Not Hispanic/Unknown) | 3/75/0 | 5/54/1 | $\chi_{2}^{2}$ = 2.62, p = 0.27 |
| Medicated/Drug-Free/ Drug-Naïve | 51/14/13 | - |  |
| **WASI** |  |  |  |
| FULL IQ | 105 (75-132, 13.8) | 109 (90-143, 9.3) | **t_136_ =** **2.03, p = 0.045,**  **g = 0.34** |
| MATRIX TS | 54 (32-68, 8.8) | 58.7 (45-70, 5.4) | **t_136_ = 3.82, p < 0.001,**  **g = 0.63** |
| VOCAB TS | 51.8 (32-73, 9.8) | 52 (34-76, 7.3) | t_136_ = 0.2, p = 0.84, g = 0.03 |
| **MCCB** |  |  |  |
| Attention/Vigilance | 38.4 (11-60, 11.8) | 47.3 (26-64, 8.5) | **t_136_ = 5.07, p < 0.001,**  **g = 0.85** |
| Speed of processing | 40.4 (8-74, 13.8) | 51.8 (35-73, 9.3) | **t_136_ = 5.67, p < 0.001,**  **g = 0.96** |
| Working memory | 41.9 (15-75, 12.3) | 48.2 (30-74, 8.9) | **t_136_ = 3.48, p < 0.001,**  **g = 0.58** |
| Verbal learning | 43.9 (25-67, 10.3) | 53.6 (37-77, 9) | **t_136_ = 5.82, p < 0.001,**  **g = 0.99** |
| Visual learning | 40.2 (8-63, 13) | 45.8 (25-61,7.9) | **t_136_ = 3.13, p = 0.002,**  **g = 0.52** |
| Reasoning and problem-solving | 44.2 (18-63, 11.4) | 50.6 (33-65,8.1) | **t_136_ = 3.81, p < 0.001,**  **g = 0.64** |
| Social cognition | 44.5 (11-66, 11.9) | 54.1 (35-67, 7.6) | **t_136_ = 5.71, p < 0.001,**  **g = 0.95** |
| Overall | 36.8 (1-65, 14.3) | 50.1 (28-71, 8) | **t_136_ = 6.89, p < 0.001,**  **g = 1.15** |
| **SAPS** |  |  |  |
|  | 6.2 (0-22, 5.9) | - |  |
| Delusions | 10.8 (0-29, 7.3) | - |  |
| Bizarre Behavior | 2.2 (0-17, 2.9) | - |  |
| Positive Formal Thought Disorder | 3.5 (0-20, 4.7) | - |  |
| **SANS** |  |  |  |
| Affective Flattening or Blunting | 8.9 (0-29, 7) | - |  |
| Alogia | 3.2 (0-12, 3.3) | - |  |
| Avolition-Apathy | 7 (0-14, 4) | - |  |
| Anhedonia-Asociality | 8.3 (0-22, 6) | - |  |
| Attention | 3.7 (0-12, 3.3) | - |  |
| **GAS** | 48.5 (23-90, 15) | - |  |

**Table S2. Descriptive and univariate analysis of sociodemographic, cognitive, and clinical variables in FEP medicated and FEP unmedicated.** Continuous variables are reported in the format mean (range, standard deviation) and underwent a Welch’s t-test, and Hedges’g was computed. For the categorical variables, a χ^2^ test was performed.

|  | **FEP medicated** | **FEP unmedicated** | **Test-statistic** |
| --- | --- | --- | --- |
| **n** | 51 | 27 |  |
| Age (years) | 22.7 (13-36, 5.1) | 23.1 (16-33.4, 4.6) | t_59_ = -0.38, p = 0.71, g = -0.09 |
| Sex (F/M) | 16/35 | 7/20 | $\chi_{1}^{2}$ = 0.25, p = 0.62 |
| Race  (Asian/Black/Mixed/Undisclosed/White) | 3/17/3/1/27 | 3/8/3/0/13 | $\chi_{4}^{2}$ = 1.94, p = 0.75 |
| Ethnicity  (Hispanic/Not Hispanic/Unknown) | 1/50/0 | 2/25/0 | $\chi_{2}^{2}$ = 1.42, p = 0.23 |
| **WASI** |  |  |  |
| FULL IQ | 105 (75-131, 14) | 107 (79-132, 13.5) | t_55_ = -0.65, p = 0.52, g = -0.15 |
| MATRIX TS | 53 (32-66, 9.1) | 56.2 (37-68, 8) | t_60_ = -1.62 p = 0.11, g = -0.37 |
| VOCAB TS | 52 (33-73, 10.1) | 51.4 (32-67, 9.5) | t_56_ = 0.26, p = 0.8, g = 0.06 |
| **MCCB** |  |  |  |
| Attention/Vigilance | 37.7 (11-60, 10.8) | 39.8 (18-60, 13.5) | t_44_ = -0.7, p = 0.49, g = -0.17 |
| Speed of processing | 40 (8-72, 13.6) | 41.1 (16-74, 15.3) | t_47_ = -0.32, p = 0.75, g = -0.08 |
| Working memory | 41.4 (15-63, 12.1) | 42.8 (22-75, 12.9) | t_50_ = -0.47, p = 0.64, g = -0.11 |
| Verbal learning | 42.9 (25-67, 10.4) | 45.9 (27-63, 10) | t_55_ = -1.26, p = 0.21, g = -0.3 |
| Visual learning | 40.6 (14-60, 12.7) | 39.4 (8-63,13.8) | t_50_ = 0.36, p = 0.72, g = 0.09 |
| Reasoning and problem-solving | 43.7 (21-63, 11.5) | 45.1 (18-61, 11.6) | t_53_ = -0.49, p = 0.62, g = -0.12 |
| Social cognition | 43.9 (11-66, 12.3) | 45.7 (14-63, 11.2) | t_58_ = -0.65, p = 0.52, g = -0.15 |
| Overall | 35.9 (1-65, 14.2) | 38.3 (2-61, 14.6) | t_53_ = -0.68, p = 0.5, g = -0.16 |
| **SAPS – symptoms description** |  |  |  |
| Hallucinations (presence/absence) | 35/16 | 22/5 | $\chi_{1}^{2}$ = 1.48, p = 0.22 |
| Delusions (presence/absence) | 49/2 | 26/1 | $\chi_{1}^{2}$ = 0.002, p = 0.96 |
| Bizarre Behavior (presence/absence) | 25/26 | 18/9 | $\chi_{1}^{2}$ = 2.22, p = 0.14 |
| Positive Formal Thought Disorder (presence/absence) | 29/22 | 10/17 | $\chi_{1}^{2}$ = 2.78, p = 0.10 |
| **SAPS (including absence of symptoms)** |  |  |  |
| Hallucinations | 5.3 (0-22, 6.1) | 7.9 (0-18, 5.3) | t_60_= -1.96, p = 0.06, g = -0.45 |
| Delusions | 10.4 (0-29, 7.5) | 11.7 (0-26, 7) | t_56_= -0.75, p = 0.46, g = -0.17 |
| Bizarre Behavior | 2 (0-17, 3.1) | 2.6 (0-8, 2.6) | t_62_= -0.87, p = 0.39, g = -0.2 |
| Positive Formal Thought Disorder | 3.6 (0-15, 4.2) | 3.4 (0-20, 5.6) | t_42_= 0.15, p = 0.88, g = 0.04 |
| **SAPS (excluding absence of symptoms)** |  |  |  |
| Hallucinations | 7.7 (2-22, 5.9) | 9.7 (3-18, 4.1) | t_54_= -1.49, p = 0.14, g = -0.38 |
| Delusions | 10.8 (2-29, 7.4) | 12.1 (2-26, 6.7) | t_55_= -0.77, p = 0.45, g = -0.18 |
| Bizarre Behavior | 4.1 (1-17, 3.4) | 3.9 (2-8, 2.2) | t_41_= 0.27, p = 0.79, g = 0.08 |
| Positive Formal Thought Disorder | 6.3 (2-15, 3.8) | 9.2 (3-20, 5.6) | t_12_= -1.53, p = 0.15, g = -0.6 |
| **SANS – symptoms description** |  |  |  |
| Affective Flattening or Blunting (presence/absence) | 42/9 | 22/5 | $\chi_{1}^{2}$ = 0.009, p = 0.92 |
| Alogia (presence/absence) | 35/16 | 16/11 | $\chi_{1}^{2}$ = 0.69, p = 0.41 |
| Avolition-Apathy (presence/absence) | 47/4 | 22/5 | $\chi_{1}^{2}$ = 1.97, p = 0.16 |
| Anhedonia-Asociality (presence/absence) | 42/9 | 22/5 | $\chi_{1}^{2}$ = 0.009, p = 0.92 |
| Attention (presence/absence) | 40/11 | 17/10 | $\chi_{1}^{2}$ = 2.15, p = 0.14 |
| **SANS (including absence of symptoms)** |  |  |  |
| Affective Flattening or Blunting | 9.2 (0-29, 7.2) | 8.4 (0-25, 7) | t_55_= 0.44, p = 0.66, g = 0.11 |
| Alogia | 3.4 (0-12, 3.4) | 2.9 (0-11, 3.3) | t_55_= 0.71, p = 0.48, g = 0.17 |
| Avolition-Apathy | 7 (0-14, 4) | 6.9 (0-13, 4.4) | t_49_= 0.11, p = 0.91, g = 0.03 |
| Anhedonia-Asociality | 8.1 (0-22, 6) | 8.6 (0-19, 6) | t_52_= -0.38, p = 0.71, g = -0.09 |
| Attention | 3.9 (0-12, 3.3) | 3.3 (0-11, 3.4) | t_60_= 0.86, p = 0.39 g = 0.2 |
| **SANS (excluding absence of symptoms)** |  |  |  |
| Affective Flattening or Blunting | 11.2 (2-29, 6.4) | 10.4 (1-25, 6.3) | t_43_= 0.48, p = 0.63, g = 0.13 |
| Alogia | 5 (1-12, 2.9) | 4.8 (1-11, 2.9) | t_29_= 0.18, p = 0.86, g = 0.05 |
| Avolition-Apathy | 7.6 (1-14, 3.5) | 8.5 (2-13, 3.1) | t_46_= -1.02, p = 0.31, g = -0.25 |
| Anhedonia-Asociality | 9.8 (2-22, 5.2) | 10.6 (2-19, 5) | t_45_= -0.59, p = 0.56, g = -0.14 |
| Attention | 5 (1-12, 2.9) | 5.2 (2-11, 2.8) | t_31_= -0.19, p = 0.85, g = -0.05 |
| **GAS** | 51 (23-90, 16.7) | 43.5 (30-75, 10) | t_72_= 2.48, p = 0.015, g = 0.55 |

**Table S3. Influence of channel array on the ROI spectral activity.**

The sample set combines two recordings from different EEG channel arrays on an Elekta Neuromag Vectorview system, using a low-impedance 10-10 system 60-channel cap. To confirm the use of those two sets in the same study, we ran a repeated-measures ANOVA on the ROIs’ absolute power spectral density (Welch’s method) in the alpha band (7–13 Hz).

The results show a p-value above the 0.05 threshold, indicating that the montage did not influence ROI estimation. The two montages can be found in the OpenNeuro database ^2,3^.

| **Array 1** | | | | **Array 2** | | | |
| --- | --- | --- | --- | --- | --- | --- | --- |
| Fp1 | C2 | F8 | P3 | Fp1 | C2 | F8 | P3 |
| Fpz | C4 | FT9 | P1 | Fp2 | C4 | FT7 | Pz |
| Fp2 | C6 | FT7 | Pz | AF7 | C6 | FC5 | P4 |
| AF7 | T8 | FC5 | P2 | AF3 | T8 | FC3 | P6 |
| AF3 | T10 | FC1 | P4 | AFz | TP7 | FC1 | P8 |
| AF4 | TP9 | FC2 | P6 | AF4 | CP5 | FCz | P10 |
| AF6 | TP7 | FC6 | P8 | AF8 | CP3 | FC2 | PO9 |
| F7 | CP3 | FT8 | PO7 | F7 | CP1 | FC4 | PO7 |
| F5 | CP1 | FT10 | PO3 | F5 | CP2 | FC6 | PO3 |
| F3 | CP2 | T9 | PO4 | F3 | CP4 | FT8 | PO4 |
| F1 | CP4 | T7 | PO8 | F1 | CP6 | T7 | PO8 |
| Fz | TP8 | C5 | O1 | Fz | TP8 | C5 | PO10 |
| F2 | TP10 | C3 | Oz | F2 | P9 | C3 | O1 |
| F4 | P7 | C1 | O2 | F4 | P7 | C1 | Oz |
| F6 | P5 | Cz | Iz | F6 | P5 | Cz | O2 |

| **Between Subjects Effects** | | | | | | | | | | | |
| --- | --- | --- | --- | --- | --- | --- | --- | --- | --- | --- | --- |
| **Cases** | | **Sum of Squares** | | **df** | | **Mean Square** | | **F** | | **p** | |
| type |  | 3.737×10^-17^ |  | 1 |  | 3.737×10^-17^ |  | 5.920 |  | 0.016 |  |
| Array |  | 1.062×10^-17^ |  | 1 |  | 1.062×10^-17^ |  | 1.682 |  | 0.197 |  |
| type × Array |  | 2.020×10^-17^ |  | 1 |  | 2.020×10^-17^ |  | 3.200 |  | 0.076 |  |
| Residuals |  | 8.460×10^-16^ |  | 134 |  | 6.313×10^-18^ |  |  |  |  |  |
|  | | | | | | | | | | | |
| *Note.* Type III Sum of Squares | | | | | | | | | | | |

**Appendix 2. Graph Theory measures**

We measured the strength (overall connectivity), clustering coefficient (functional segregation), and path length (functional integration) of each subject’s graph at each frequency band using the Brain Connectivity Toolbox (Rubinov and Sporns, 2010).

***Strength***

Node strength is the sum of the weights of links connected to the node.

$$str=\sum(CIJ)$$

where CIJ is the undirected weighted connection matrix, and str is the node strength.

***Clustering coefficient***

On the other hand, the clustering coefficient is a measure of functional segregation. The clustering coefficient is the likelihood that neighbors of a node are connected and indicates the extent of local interconnectivity or cliquishness in a network.

$$C^{W}=\frac{1}{n}\sum_{i∊N} \frac{2t_{i}^{w}}{k_{i}(k_{i}-1)}$$

where $t_{i}^{w}$ is (Weighted) geometric mean of triangles around *i* and $k_{i}$ is the Degree of a node *i*.

***Characteristic path length***

The path length measures the shortest route (regarding edges traversed) between two nodes. It is commonly referred to as a measure of functional integration. The average shortest path length, or characteristic path length, is the average shortest path length between all pairs of nodes in the network (Rubinov and Sporns, 2010).

$$L^{W}=\frac{1}{n}\sum_{i∊N} \frac{\sum_{j∊N,j\neq i} d_{ij}^{w}}{n-1}$$

where N is the set of all nodes in the network, n is the number of nodes, $d_{ij}^{w}$ is the shortest weighted path length between i and j.

***Edgewise index***

We used a metric called network index (edgeNI), used in Yassine et al.^4^, as the average weight (connectivity) of the output significant networks issued from NBS:

edge𝑁𝐼=∑𝑁𝑖𝑊𝑖𝑁,

where 𝑊𝑖 represents the connectivity value of the edge i of the significant network from NBS, and N represents the total number of edges in this network.

**Appendix 3. Microstate-derived measures**

***Global connectivity power*** (GCP): defined as the standard deviation of all connectivity indices at a given time point, providing a measure of connectivity strength analogous to global field power (GFP) (Murray et al. 2008).

***Individual explained variance:*** the proportion of GCP explained by each BNS, indicating how well each state represents the data:

$$iEV_{n}=Corr\left( x_{n},a_{l_{n}} \right)^{2}\cdot\frac{GCP_{n}^{2}}{\sum_{n^{'}}^{N} GCP_{n^{'}}^{2}}$$

where x_n_ is the n^th^ sample of the dynamic connectivity tensor of the subject, a_ln_ is the BNS connectivity matrix, and GCP is the computed global connectivity power. The individual explained variance of a subject is the average of the explained variances of its sample.

***Coverage:*** the proportion of recording time during which a BNS is the dominant active state.

$$Coverage_{k}=\frac{N_{k}}{N_{total}}$$

Where N_k_ is the number of matrices assigned to BNS k, and N_total_ is the total number of matrices.

***Duration:*** the average time a BNS remains active before transitioning to another state

$$Duration_{k}=mean\left( L_{k} \right)$$

Where L_k_ represents the lengths of segments assigned to BNS k

***Occurrence:*** the number of distinct episodes in which a specific BNS appears.

**Table S4 – List of clustering criteria for the Meta-criterion**

**Criterion**

Krzanowski-Lai criterion

Normalised Krzanowski-Lai criterion

Cross-validation criterion

Calinski-Harabasz index

Enhanced Davies-Bouldin index

PBM index

S_Dbw validity index

Score Function index

SV index

Xie-Beni index

WB index

**Appendix 4. Meta-criterion computation**

The following process combined the 11 criteria into a single overarching metric, following the approach of *Custo et al*. ^5^. In the first step, each of the 11 criteria was ranked separately by sorting the values from lowest to highest and using the relative positions. Then, the criteria were merged using a formula designed to achieve two key goals: maximize average ranking and favor unanimity - having all 11 criteria in close agreement. To do so, maximizing the signal-to-noise ratio across criteria is defined as achieving maximum unanimity.

This leads to the following equations, defined by Custo et al.:

$MetaCriterion = IQM \cdot SNR$ (Eq: 1)

with IQM = Interquartile Mean (Criteria) (Eq: 2)

IQR = Interquartile Range (Criteria) (Eq: 3)

$SNR = IQM/IQR$ (Eq: 4)

which finally gives:

$MetaCriterion=IQM^{2}/IQR$ (Eq: 5)

The best number of clusters n according to the Metacriterion is then:

arg_n_ max_n≥4_ [*MetaCriterion*(n)] (Eq: 6),

meaning that the optimal number of clusters is at the peak of the meta-criterion, where the average of all criteria is high, and the dispersion is low. The x-axis is the number of clusters, and the y-axis is the normalized criterion values, ranging from 0 to 1.

**Figure S11. Criteria (A) and metacriterion (B) results applied to the cluster solution.** The optimal number of clusters is at the peak of the meta-criterion, where the average of all criteria is high, and the dispersion is low. The x-axis is the number of clusters, and the y-axis is the normalized criterion values, ranging from 0 to 1.

**Figure S12. List of ROIs and RSN.** AUD: Auditory Network; DAN: Dorsal Attentional Network;
DMN: Default Mode Network; MOT: Motor Network; SAN: Salient Network; VIS: Visual Network

**References**

1.	Zalesky, A., Fornito, A. & Bullmore, E. T. Network-based statistic: Identifying differences in brain networks. *NeuroImage* **53**, 1197–1207 (2010).

2.	Salisbury, D., Seebold, D. & Coffman, B. EEG: First Episode Psychosis vs. Control Resting Task 1. [object Object] https://doi.org/10.18112/OPENNEURO.DS003944.V1.0.1 (2022).

3.	Salisbury, D., Seebold, D. & Coffman, B. EEG: First Episode Psychosis vs. Control Resting Task 2. [object Object] https://doi.org/10.18112/OPENNEURO.DS003947.V1.0.1 (2022).

4.	Yassine, S. *et al.* Functional Brain Dysconnectivity in Parkinson’s Disease: A 5‐Year Longitudinal Study. *Mov. Disord.* **37**, 1444–1453 (2022).

5.	Custo, A. *et al.* Electroencephalographic Resting-State Networks: Source Localization of Microstates. *Brain Connect.* **7**, 671–682 (2017).
